# The Autophagy Machinery Contributes to E-cadherin Turnover in Breast Cancer

**DOI:** 10.3389/fcell.2020.00545

**Published:** 2020-06-30

**Authors:** Valentina Damiano, Paola Spessotto, Giulia Vanin, Tiziana Perin, Roberta Maestro, Manuela Santarosa

**Affiliations:** ^1^Unit of Oncogenetics and Functional Oncogenomics, Centro di Riferimento Oncologico di Aviano (CRO) IRCCS, Aviano, Italy; ^2^Unit of Molecular Oncology, Centro di Riferimento Oncologico di Aviano (CRO) IRCCS, Aviano, Italy; ^3^Pathology Unit, Centro di Riferimento Oncologico di Aviano (CRO) IRCCS, Aviano, Italy

**Keywords:** autophagy, E-cadherin, breast cancer, SQSTM1, adherens junctions

## Abstract

Autophagy is an intracellular catabolic process that is increasingly being recognized as a crucial factor in several human diseases including cancers. Mounting evidence suggests that autophagy allows tumor cells to overcome otherwise fatal stresses and to increase dissemination. Nevertheless, how autophagy controls these processes and in particular how it impinges on cell-cell adhesion is still poorly understood. Here, we investigate the role of autophagy in the turnover of the epithelial adhesion molecule E-cadherin in the context of breast cancer. We demonstrated in breast cancer cell lines that autophagy impinges on E-cadherin expression and in the configuration of adherens junctions. Besides, we showed that E-cadherin colocalizes with LC3B and SQSTM1/p62, two components of the autophagosome machinery. Pull down and immunoprecipitation analyses provided evidence that E-cadherin and SQSTM1/p62 physically interact. Moreover, the physical closeness of E-cadherin and SQSTM1/p62 was demonstrated by proximity ligation assays in breast cancer cell lines and primary tumors. Finally, we proved that the silencing of SQSTM1/p62 diminished the E-cadherin/LC3B colocalization, further supporting the role of SQSTM1/p62 in E-cadherin delivery to autophagosomes. These findings suggest that the activation of autophagy, reported in breast cancers with poor prognosis and in dormant breast cancer cells, may contribute to the control of tumor progression via downmodulation of E-cadherin protein levels.

## Introduction

Autophagy is an evolutionarily conserved catabolic process whereby a double membrane compartment, named autophagosome, sequesters cytoplasmic cargo and delivers it to lysosomes for degradation (Mizushima and Komatsu, 2011). Dysfunction of autophagy is involved in multiple human diseases (Levine and Kroemer, 2008; Liang, 2010), including cancer where autophagy plays a double role: in oncogenically challenged cells autophagy may prevent tumor progression by removing ROS and damaged mitochondria (White, 2012; Amaravadi and Debnath, 2014); on the other hand, in overt tumors, autophagy seems to contribute to tumor cell survival by promoting nutrient recycling and sustaining anti-apoptotic pathways (Levy et al., 2017; Mowers et al., 2017). Along with this line, increased autophagic flux has been reported for various types of tumors, including breast cancer (BC) (Lazova et al., 2012; Mikhaylova et al., 2012; Giatromanolaki et al., 2018).

Mounting evidence suggests that autophagy endows tumor cells with the capability to survive anoikis, hypoxia and chemotherapy treatments (Levine and Kroemer, 2008; Levy et al., 2014; Daskalaki et al., 2018). Besides, in certain contexts, autophagy has been shown to trigger an epithelial to mesenchymal transition-like program that accounts for the downregulation of cell-cell and cell-basal membrane interactions and an increase in migratory capability (Nieto et al., 2016; Mowers et al., 2017; Alizadeh et al., 2018). These phenomena contribute to cancer stem-like cell maintenance and high metastatic potential of BC, in particular of triple negative BC (ER, PGR, and HER2 receptor negative tumors) (Vera-Ramirez, 2019).

E-cadherin is a major component of the adherens junctions that maintain cell-cell adhesion, basal-apical polarity, and epithelial tissue homeostasis, thus restraining cell motility and cancer progression (Perl et al., 1998; Izaguirre and Casco, 2016; Kourtidis et al., 2017; Carneiro et al., 2019). E-cadherin interacts with p120 catenin and beta-catenin through its cytoplasmic domain forming a core complex that connects the adherens junctions to the actin cytoskeleton and preserves junctional maintenance and dynamics (Ireton et al., 2002; Brüser and Bogdan, 2017).

Several mechanisms have been described that contribute to the downregulation of E-cadherin in cancer. Loss of function mutations of the CDH1 gene, which result in impaired E-cadherin protein expression, are commonly detected in lobular breast carcinomas and diffuse gastric cancers (Graziano et al., 2003; Ciriello et al., 2015). In other tumors, the CDH1 gene is transcriptionally repressed (Nieto et al., 2016; Kourtidis et al., 2017). Augmented endocytosis of E-cadherin has also been reported (Jones et al., 2006; Kourtidis et al., 2017) and autophagy-induced degradation, due to overexpression of SIRT1, SPHK1, or PHF8 have been described in melanoma and hepatocarcinoma (Liu et al., 2017; Sun et al., 2018; Zhou et al., 2018). Yet, the mechanisms whereby autophagy impacts on E-cadherin expression in the setting of BC are not fully elucidated.

Here we demonstrate that the autophagy machinery impinges upon E-cadherin protein levels by chaperoning it to autophagosome via interaction with the autophagic cargo adaptor SQSTM1/p62.

## Materials and Methods

### Cell Models and Treatments

The human breast cancer cell lines MDA-MB-231 (here referred to as MDA231), HCC1937, and MCF7 were obtained from the ATCC (LGC Standards). All cell lines were periodically authenticated by short tandem repeat profiling and tested mycoplasma-negative. They were cultured as previously described (Santarosa et al., 2009; Borgna et al., 2012) or, also, in Hanks’ Balanced Salt Solution (HBSS; Sigma-Aldrich) for achieving nutrient starvation; in medium containing either 50 μM Chloroquine Diphosphate Salt (CQ; Sigma-Aldrich) or 40 nM Bafilomycin A1 (BAF; Bioviotica Naturstoffe) to block autophagy by preventing autophagosome acidification and degradation; in medium containing 5 μM Rapamycin (Rapa; LC Laboratories), an inhibitor of mTOR, which activates autophagy or in the presence of 5 mM 3-Methyladenine (3MA; Selleckchem) to inhibit autophagosome formation.

Stably ATG7 (shATG7) and SQSTM1/p62 (shp62-1, shp62-2) silenced cell lines, as well as control (shCTR) cells were generated by using lentiviral plasmids obtained from a modified version of pRSI9 DECIPHER vector (Cellecta) as previously described (di Gennaro et al., 2018). Individual short hairpin RNA (shRNA) sequences are reported in Supplementary Table 1.

To generate MDA231-GFP-LC3B and HCC1937-GFP-LC3B cell lines, the GFP-LC3B sequence was amplified form pEX-GFP-hLC3WT (Addgene #24987; Tanida et al., 2008) with the following primers: F 5′-AAAAAACCGGTATGGTGAGCA AGGGGGAGG and R 5′-AAAAAGAATTCCACCTGAGGAGT GAATTGAGC. The GFP-LC3B PCR was cloned, as an AgeI-EcoRI fragment, into a pLJM-based vector (Addgene #19319; Sancak et al., 2008) and MDA231 and HCC1937 cells were then lentivirally infected. pLJM-EGFP was used as a negative control.

MDA231-E-cad-Strep-Tag and MDA231-p62-Strep-Tag cells were generated by transduction with either pLJM-Ecad-Strep-Tag or pLJM-p62-Strep-Tag lentiviral vectors. To generate vectors, we used the Strep-tag sequence, which exhibits intrinsic affinity toward Strep-Tactin and is codified by the pPSG-IBA105 vector (IBA LifeSciences), and firstly cloned in the pCS2-myc vector (a gift from Dave L Turner). The engineered vector was used as a template for the Strep-tag amplification by using the following primers F 5′-AAAACCGGTACCATGGGTTAACGTTAGCGC ATGGAGTCATCC and R 5′-AAAAGAATTCCTCGAGTTAA TTCAAGTCCTCTTCAG designed to keep out the Strep-tag ATG site, to insert a TAA stop codon at the 3′ terminus, to insert restriction sites AgeI at 5′ site and EcoRI at 3′ site and an in-frame HpaI site in the 5′ side of Strep-tag. After AgeI/EcoRI digestion the amplified sequence was cloned in the pLJM-EGFP vector (Addgene #19319; Sancak et al., 2008). The generated pLJM-Strep-Tag vector was then used as a backbone to clone E-cadherin (at N terminus of Strep-tag). This was achieved by amplifying the gene from the E-cadherin-GFP plasmid (Addgene #28009; Miranda et al., 2001) with oligos allowing for NheI/HpaI digestion (F-5′ AAAAAgctagcTATCGAATTCCGGAAAGCAC and R 5′ AAAAAGTTAACGCTCCGCGGGTCGTCCTC). To generate the pLJM-p62-Strep-Tag vector, p62 was first obtained by HindIII and NotI digestion from HA-p62 plasmid (Addgene #28027; Fan et al., 2010) and cloned in pcDNA 3.1. The cloned fragment was then digested with ApaI, blunted by incubating with DNA Polymerase I Klenow Fragment (New England Biolabs), digested with NheI and cloned in the pLJM-Strep-Tag.

MDA231 cells were engineered to ectopically express E-cadherin (MDA231-E-cad) by transduction of the pLJM-E-cad lentiviral vector. This vector was obtained from pLJM-Ecad-Strep-Tag by removing the Strep-Tag sequence. pLJM-EGFP vector was used as a negative control.

All the generated vectors were verified by Sanger sequencing. Lentiviral particles were generated as previously described (Damiano et al., 2017). Puromycin treatment selected the efficiently transduced cells.

### Western Blot Analyses

Protein extraction and western blot were performed as previously described (Santarosa et al., 2010). Supplementary Table 2 reported the primary antibodies used. Immunoreactivity was detected with anti-mouse or anti-rabbit secondary antibodies HRP-labeled (Perkin Elmer) and Western Lightning Chemiluminescence Reagent Plus (Perkin Elmer). Chemidoc XRS+ system (Bio-Rad) and ImageLab imaging software (Bio-Rad) were used to capture and analyze images, respectively. Immunoreactivity to E-cadherin and relative loading control, in HCC1937 and MCF7 cell lines, was measured by using Alexa Fluor-680 conjugated secondary antibodies and the Odyssey infrared imaging system (LI-COR, Biosciences) for blot visualization and quantization. Results were confirmed in at least 3 independent experiments.

### Immunofluorescence

For immunofluorescence analyses, cells were cultured on coverslips in the medium described in the text. After treatment, cells were fixed in 4% paraformaldehyde/PBS for 15 min, permeabilized with 0.4% Triton X-100/PBS for 10 min, and incubated for 1 h with 0.04% Triton X-100/PBS containing 3% of BSA to block non-specific antigens. In the case of antibody-mediated LC3B labeling, cells were permeabilized with ice-cold 100% methanol. Cells were stained with the appropriate antibody (reported in Supplementary Table 2) at 4°C overnight. Primary antibodies were visualized with goat anti-mouse Alexa Fluor 594 or 633 (Thermo Fisher Scientific), or with goat anti-rabbit Alexa Fluor 594 or 633 (Thermo Fisher Scientific). TO-PRO-3 (Thermo Fisher Scientific) was used for nuclear labeling. Cells were then analyzed by using the TCS-SP8 Confocal System (Leica Microsystems) interfaced with the Leica Application Suite (LAS) software. When all three fluorochromes were used, individual cells were identified by capturing transmitted light images. Total and colocalized dots were quantified by using the Fiji/ImageJ software (Schneider et al., 2012) and the ComDet v.0.5.1 plugin^1^.

### Analyses of Adherens Junctions

Cells were seeded on coverslips and grown to confluence, then cells were either treated with standard medium, starved for 8 h, or treated with 5 μM Rapamacyn for 24 h or 10 mM 4-dithiothreitol (DTT; Sigma-Aldrich) for 3 h. Immunofluorescence staining was then carried out as above described. Alexa Fluor 488 or 594 Phalloidin (Thermo Fisher Scientific) was used to probe F-actin. Images were captured with TCS-SP8 Confocal System (Leica Microsystems) and analyzed with Fiji/ImageJ software (Schneider et al., 2012), similarly, to what previously described (Di Russo et al., 2017). Briefly, adherens junctions, considered as the E-cadherin/beta-catenin positive cell-cell border fragment, were measured on the focal plane corresponding to the maximum intensity of the E-cadherin signal. Adherens junctions ratio was calculated as the length of adherens-junctions over the length of the perimeter of the cell that was manually measured by using the border of phalloidin maximum intensity projections. Experiments were performed in triplicate and, overall, at least 100 cells per sample were measured.

Time-lapse images of cells were captured every 20 min for 20 h by AF6000 microscope system and LASX software (Leica Microsystems).

### Patients

Formalin-fixed, paraffin-embedded specimens of 18 consecutively E-cadherin expressing BC were prospectively collected at the CRO Aviano National Cancer Institute. E-cadherin expression was routinely assessed at the Pathology Unit of the Institute. Informed consent was obtained and the use of patient samples was approved by the Institutional Review Board. Clinicopathological data were retrieved from clinical records.

### Proximity Ligation Assay (PLA)

For *in vitro* PLA experiments, cells were cultured on coverslips, treated as indicated in the text and fixed and permeabilized as reported in the immunofluorescence section.

For *ex vivo* analyses, 5 μm breast cancer tissue sections were deparaffinized by incubation in xylene (soaking twice for 10 min each), and rehydrated with ethanol solutions (twice in 100, 90, 70, and 50% ethanol solutions for 3 min each) and water (twice for 5 min). Heat-induced antigen retrieval was performed in citrate buffer (10 mM, pH 6) for 10 min in the microwave. Permeabilization was carried out as reported above. Tissues were treated for 10 min with 0.1M Glycine to reduce background fluorescence. Slides were checked for the emission of autofluorescence. Indeed, four breast cancer tissues showed red- and green-emitting dots in the cytoplasm of tumor cells that might reflect the presence of lipofuscins, fluorescent components that accumulate in the lysosomal compartment of many cell types (Moreno-García et al., 2018; Supplementary Figure 1A). These samples were excluded from further investigations.

Then, the analysis was performed by using the DuoLink PLA kit (Sigma-Aldrich) with Detection Reagents Red and following the manufacturer’s protocol. Supplementary Table 2 displayed the antibodies used in the analyses. Coverslips/slides were incubated overnight at 4°C with primary antibodies and 1 h and 30 min at 37°C with secondary antibodies-PLA probes. After that, ligation and amplification steps were performed as the producer instructions. Finally, coverslips or slides were mounted with DuoLink *in situ* mounting medium containing DAPI. Images were captured with the Nikon Eclipse-Ti fluorescence microscope equipped with Plan Fluor 40x objective and with TRITC and UV2A filter cubes (EX-filter 540/25, barrier-filter 590LP; EX-filter 355/50, barrier-filter 410, respectively).

For dot quantification, we analyzed the captured images with Fiji/ImageJ software (Schneider et al., 2012) and ComDet v.0.3.7 plugin for FIJI with a particle size of 4 pixels and signal-to-noise ratio 4. At least 50 cells per sample were analyzed.

### Strep-Tag Pull-Down and Immunoprecipitation Assays

MDA231-E-cad-Strep-Tag and MDA231-p62-Strep-Tag cells were lysed with Pierce Lysis Buffer. Lysates were incubated with Strep-Tactin Sepharose 50% suspension (IBA Lifesciences) and, after washing, bound proteins were retrieved with Laemmli buffer and analyzed in western blot.

Immunoprecipitation (IP) of endogenous proteins was performed by lysing cells in PLB buffer (20 mM Tris pH 7.5, 200 mM NaCl, 1 mM EDTA, 0.5% Igepal) supplemented with Complete Protease Inhibitor Cocktail (Sigma-Aldrich) and PMSF. Protein lysates (0.5 mg) were immunoprecipitated overnight with the mouse anti-SQSTM1/p62 antibody (Santa Cruz Biotechnology) and then conjugated to Protein G Sepharose 4 Fast Flow beads (Sigma-Aldrich) for 2 h. Immunocomplexes were washed five times with PLB buffer, resuspended in Laemmli buffer containing β-mercaptoethanol and heated at 100°C for 10 min prior gel loading. Proteins were resolved by SDS–PAGE (4–15% gradient). E-cadherin and SQSTM1/p62 were immunodetected with the mouse anti-E-cadherin (BD Biosciences) and the rabbit anti-SQSTM1/p62 (Thermo Fisher Scientific) antibodies, respectively.

### Statistical Analyses

Statistical differences between groups were evaluated using a one-way analysis of variance (ANOVA) followed by a test for linear trend or by unpaired *t*-test, taking advantage of GraphPad PRISM8 software (GraphPad Software). Differences in the PLA puncta per cell between samples were appraised by using the Mann–Whitney Rank Sum Test.

## Results

### Autophagy Modulation Affects E-cadherin Expression in Breast Cancer

**FIGURE 1:**
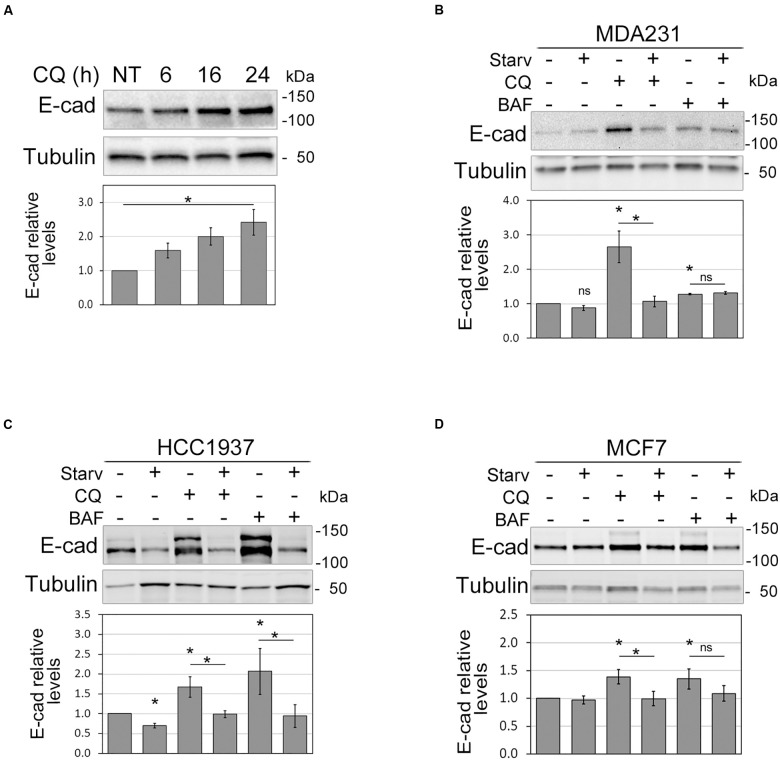
Autophagy modulation affects E-cadherin levels. **(A)** Immunoblot showing the increment of E-cadherin (E-cad) levels in MDA231 cells treated with CQ for 6, 16, and 24 h. NT represents the untreated cells. γ-tubulin (Tubulin) was used as a loading control. *Statistical significance (*p* < 0.01) in the ANOVA test followed by a test for linear trend. **(B–D)** Representative immunoblots displaying the levels of E-cadherin (E-cad) in MDA231 **(B)**, HCC1937 **(C)**, and MCF7 **(D)** cell lines either treated with CQ or BAF (for 8 h), in standard medium or upon nutrient starvation (Starv). Graphs below blots report the mean of E-cadherin relative levels of three experiments with SEM as error bars. E-cadherin relative levels were obtained by normalization over γ-tubulin (Tubulin, loading control) and rescaling to the untreated sample. The asterisks above the histograms and lines mean statistical significance (*p* < 0.05) in the unpaired *t*-test comparisons to the untreated sample or between indicated samples, respectively. ns, not statistically significant.

To shed light on the relationship between autophagy and E-cadherin expression in the setting of BC, we inhibited autophagosome acidification and digestion by chloroquine (CQ) in MDA231, a breast cancer cell line that expresses a very low level of E-cadherin (Borgna et al., 2012). CQ treatment produced a significant time-dependent augment of E-cadherin within 24 h, corroborating the hypothesis of a role for autophagy in E-cadherin regulation in these cells (Figure 1A and Supplementary Figure 2A). To further explore this hypothesis, autophagy was induced by starvation and/or inhibited with either CQ or Bafilomycin A1 (BAF, which prevents autophagosome acidification) in MDA231 as well as in HCC1937 and MCF7 E-cadherin expressing BC cell lines. The efficacy of treatments was monitored by assessing the expression of the un-conjugated and the phosphatidylethanolamine (PE) conjugated forms of MAP1LC3B (LC3B), LC3B-I, and LC3B-II, respectively (Supplementary Figures 2B–D). In fact, short after synthesis LC3B is cleaved to generate LC3B-I that, upon autophagy activation, is PE-conjugated to form LC3B-II. This, in turn, is recruited onto the growing phagophore and therein degraded (Klionsky et al., 2016). As expected, the ratio between LC3B-II and LC3B-I augmented in starved samples and in cells in which autophagosome degradation was blocked. SQSTM1/p62, an autophagy receptor that interacts with autophagic substrates and LC3B and that is itself an autophagic target (Johansen and Lamark, 2011), diminished upon starvation and accumulated when autophagy was blocked (Supplementary Figures 2B–D). The magnitude of autophagy modulation varied in the different cell models, a finding that is keeping with the reported differences in the response to autophagy of breast cancer cell lines (Maycotte et al., 2014; Zhu et al., 2017).

Notably, in MDA231, HCC1937, and MCF7 the inhibition of autophagy induced by CQ and BAF was paralleled by an augment of E-cadherin protein levels; vice versa, starvation reduced E-cadherin levels in HCC1937 and counteracted the effect of autophagy inhibitors (Figures 1B–D). In HCC1937 and MCF7 cell lines, the increased levels of E-cadherin associated to the inhibition of autophagosome lysis rendered more visible the anti-E-cadherin reactive band of higher molecular weight. This extra band might represent uncleaved pro-E-cadherin or post-translationally modified (e.g., glycosylation, ubiquitination) E-cadherin forms (Geng et al., 2012; Carvalho et al., 2016).

On the other hand, treatment of MDA231 with 3-Methyladenine, an inhibitor of autophagosome formation, produced an increment of E-cadherin at the membrane levels (Supplementary Figures 3A,B).

Thus, these data support a role for autophagy in the control of E-cadherin expression.

### Autophagy Induction Disrupts Cell-Cell Adhesion

Since E-cadherin is pivotal in maintaining epithelial features, we then explored whether autophagy modulation, by affecting E-cadherin levels, impinges on cell morphology and cell-cell junctions.

**FIGURE 2:**
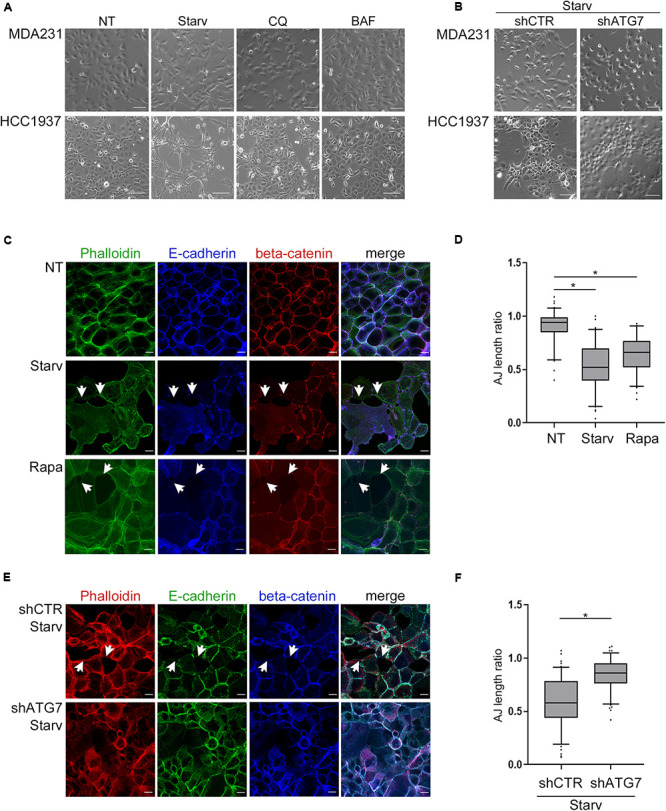
Autophagy induction impinges upon cell morphology and cell-cell adhesion. **(A)** Depicting images (bright field) of MDA231 and HCC1937 untreated (NT), starved (Starv; 8 h) or treated with either CQ or BAF (8 h). Scale bar, 50 μm. **(B)** Representative images of MDA231 and HCC1937 cell models silenced for ATG7 (shATG7) and the respective controls (shCTR). Cells were starved for 8 h. Scale bar, 50 μm. **(C)** Confocal fluorescence images of HCC1937 untreated (NT), starved (Starv) or treated with 5 μM Rapamycin (Rapa) for 24 h and immunostained with Alexa Fluor 488 Phaolloidin, E-cadherin/Alexa Fluor 633 (E-cad) and beta-catenin/Alexa Fluor 594. White arrowheads indicate representative loss of cell-to-cell cohesion. Scale bar, 20 μm. **(D)** Quantification of adherens junctions (AJ length ratio; measured as described in section “Materials and Methods”) of the HCC1937 cells treated as described in **(C)**. **(E)** Confocal fluorescence images of HCC1937 cells silenced for ATG7 (shATG7) and the related control (shCTR) starved for 8 h. Cells were immunostained with E-cadherin/Alexa Fluor 488 (E-cad), beta-catenin/Alexa Fluor 633 and Alexa Fluor 594 Phaolloidin to probe F-actin. White arrowheads indicate representative lack of cell-to-cell cohesion regions. Scale bar, 20 μm. **(F)** Quantification of AJ length ratio of the cells described in **(E)**. **(D,F)** At least 100 cells per sample were measured in three independent experiments. Lines within the boxes mark the median, boundaries represent the 25th and the 75th percentiles, whiskers below and above the boxes indicate the 5th and 95th percentiles, respectively, and dots the outliers. An unpaired *t*-test was used to assess statistical significance. *p < 0.001.

Indeed, treatments with either CQ or BAF promoted a cobblestone-like morphology in the spindle-like MDA231 cells whereas the starvation-mediated induction of autophagy increased the spindle-like features of both MDA231 and HCC1937 models (Figure 2A). Notably, the block of autophagy by silencing of ATG7, a key component of the autophagic machinery, neutralized the effects on cell morphology induced by starvation, a phenomenon particularly evident in HCC1937 cells which, compared to MDA231, display higher levels of E-cadherin and cell-to-cell adhesion (Figure 2B and Supplementary Figures 4A,B). In these same cells, the induction of autophagy by starvation or Rapamycin treatment (Supplementary Figure 4C) resulted in a decrease of E-cadherin and beta-catenin staining at cell-cell borders, associated with a significant drop in the cell-to-cell adhesion (Figures 2C,D). As a positive control, we used cells treated with 1,4-dithiothreitol (DTT), a reagent known for reducing the disulfide bridges that sustained the E-cadherin connection between neighboring cells (Brückner and Janshoff, 2018; Supplementary Figure 4D). Moreover, starvation, similar to DTT, produced a remarkable disruption of cell monolayer (Supplementary time-lapse videos).

Again, the silencing of ATG7 abated the starvation-mediated decrement of adherens-junctions length ratio (Figures 2E,F) supporting the role of autophagy in these phenomena.

### E-cadherin Localizes in Autophagosomes

**FIGURE 3:**
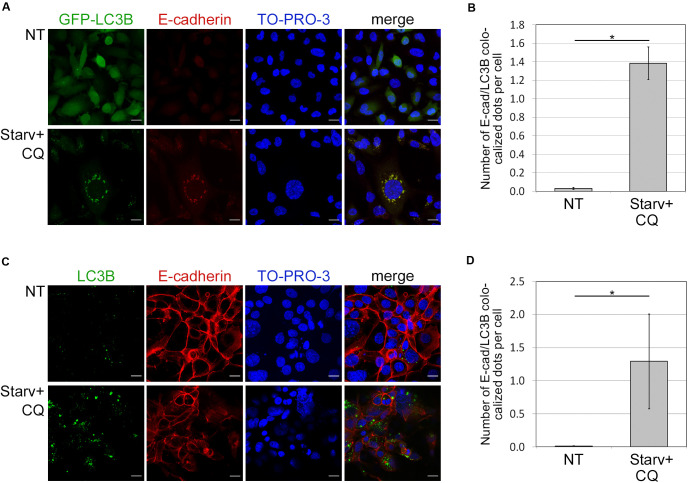
Colocalization of E-cadherin and the autophagosomal LC3B. **(A)** MDA231 cells expressing GFP tagged form of LC3B (GFP-LC3B) were either grown in standard conditions (NT) or starved in the presence of CQ (Starv+CQ) for 8 h. Cells were stained with anti-E-cadherin/Alexa594 antibodies and analyzed by confocal microscopy. Nuclei were labeled with TO-PRO-3. Representative merge images are reported. **(B)** Quantification of GFP-LC3B and E-cadherin colocalized dots per cell measured in the experiments as in **(A)**. The analysis was performed as described in section “Materials and Methods.” **(C)** HCC1937 cells treated as in **(A)** and stained with anti-LC3B/Alexa488, anti-E-cadherin/Alexa549 antibodies, and TO-PRO-3. **(D)** Graph reported the quantification of the experiments carried out as in **(C)**. **(B,D)** At least 100 cells per sample were measured in three independent experiments. Graphs report the mean number of E-cadherin and LC3B colocalized dots per cell of at least 100 cells per sample measured in three independent experiments. Bars represent SEM; *p < 0.01 in unpaired *t*-test.

Because autophagy is known to deliver protein aggregates to lysosomes for degradation, we investigated whether E-cadherin was a target of such a metabolic mechanism. To this end, we measured the colocalization of E-cadherin with LC3B dots, as previously described (Birgisdottir et al., 2019). E-cadherin and LC3B colocalization was detected in both HCC1937 (endogenous LC3B) as well as in MDA231-GFP-LC3B engineered to express ectopic LC3B (Figures 3A–D). Endogenous and GFP-LC3B dots were barely detectable in untreated (NT) cells because of their rapid turnover. Instead, upon starvation-induced autophagy and CQ-mediated block of the autophagosome lysis (Starv+CQ), LC3B dots became readily detectable as puncta (Figures 3A–D and Supplementary Figures 5A,B). Interestingly, in MDA231 and HCC1937 cell lines, treatments caused an increment of E-cadherin/LC3B colocalized dots (Figures 3A–D).

In both treated and untreated MDA231-EGFP negative control, EGFP appeared diffuse (Supplementary Figure 5C) discarding the possibility that puncta observed in treated cell models were the result of unspecific aggregates. Besides, GFP-LC3B and endogenous LC3B yielded similar levels of dots in the MDA231 cell line (Supplementary Figures 5A,B).

### SQSTM1/p62 Interacts With E-cadherin

SQSTM1/p62 is a multidomain protein whose UBA (Ubiquitin associated) and LIR (LC3 interacting region) domains, by interacting with ubiquitinated autophagic substrates and LC3, respectively, are crucial in targeting ubiquitinated proteins or aggregates to autophagosomes (Taniguchi et al., 2016).

**FIGURE 4:**
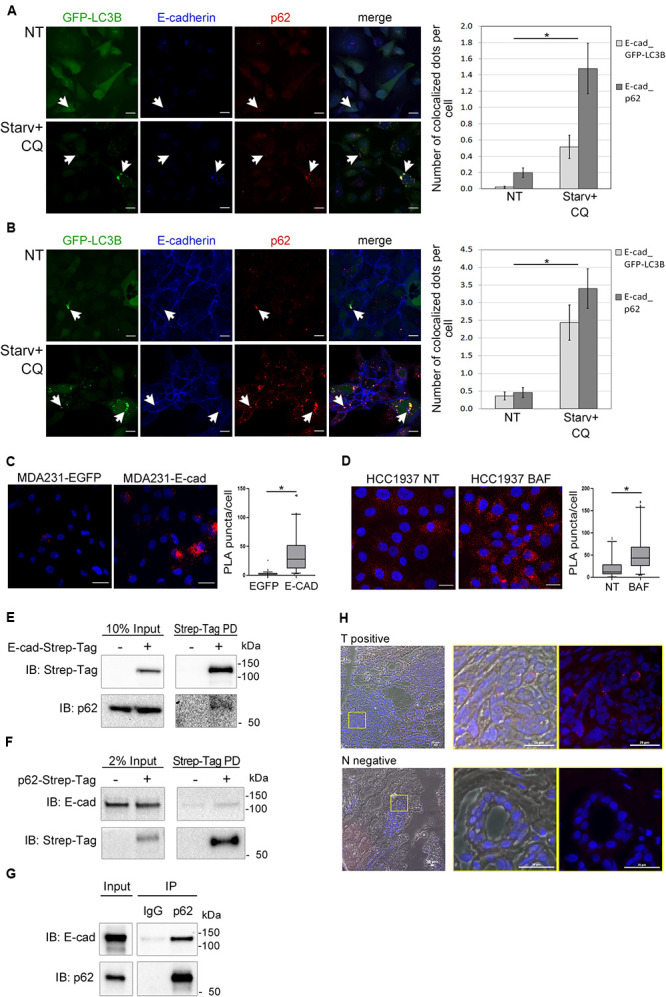
E-cadherin and SQSTM1/p62 interaction. **(A,B)** Representative confocal images of MDA231-GFP-LC3B **(A)** and HCC1937-GFP-LC3B **(B)** cell lines untreated (NT) or starved in the presence of CQ (Starv+CQ) for 8 h. Cells were stained with rabbit anti-E-cadherin/AlexaFluor 633 (E-cadherin) and anti- SQSTM1/p62/AlexaFluor 594 (p62) antibodies. Individual and merged fluorescence images are reported on the left. Arrowheads indicate representative spots of co-occurrence of GFP-LC3B, E-cadherin and SQSTM1/p62 (p62) signals. On the right, the graphs showed the mean number of dots per cell in which E-cadherin and GFP-LC3B (E-cad_GFP-LC3B) or E-cadherin and SQSTM1/p62 (E-cad_p62) colocalized. Data are the result of the quantification of at least 100 cells per sample measured in three independent experiments. Bars represent SEM; *p < 0.01 in unpaired *t*-test. **(C)** In situ proximity ligation assay (PLA) in MDA231 cells ectopically expressing E-cadherin (MDA231-E-cad) or EGFP (MDA231-EGFP as a control). Cells were stained with rabbit anti-E-cadherin and mouse anti SQSTM1/p62 antibodies. Nuclei were stained with DAPI (blue); PLA positive signals appear as red puncta. Scale bar, 20 μm. Quantification of PLA puncta/cell in at least 50 cells per sample was shown in the box plots on the right. **(D)** *In situ* PLA, carried out as in **(C)**, in HCC1937 cells treated with either BAF or DMSO (NT, as a control) for 8 h. Scale bar, 20 μm. Quantification of PLA red puncta per cell in at least 50 cells per sample is reported in the box plots on the right. **(C,D)** Lines within the boxes mark the median, boundaries represent the 25th and the 75th percentiles, whiskers below and above the boxes indicate the 5th and 95th percentiles, respectively, and dots the outliers. *p < 0.001 evaluated by using the Mann-Whitney Rank Sum Test. **(E)** Strep-Tag-mediated pull-down (PD) assay shows that a fraction of SQSTM1/p62 (p62) is collected through Strep-Tactin binding of E-cadherin Strep-Tag (Strep-Tag PD, on the right) expressed in MDA231. E-cadherin-Strep-Tag (Strep-Tag) and SQSTM1/p62 (p62) levels in the input samples are reported on the left. **(F)** Immunoblots representing the PD of p62-Strep-Tag in MDA231 cells. p62-Strep-Tag (p62) and E-cadherin (E-cad) proteins were analyzed by western blot (on the right). Input levels of the proteins are reported on the left. In **(E,F)**, cellular extracts from cells ectopically expressing Strep-Tag-only vector (left lines) were used as a negative controls. **(G)** Immunoblots representing the immunoprecipitation (IP) of endogenous E-cadherin (E-cad) from HCC1937 cell extract using the antibodies anti- SQSTM1/p62 (p62) or anti-immunoglobulin G1 (IgG), used as a negative control. Anti- E-cadherin (E-cad) and anti- SQSTM1/p62 (p62) antibodies are used for immunoblotting. Input levels of the proteins are reported on the left. **(H)** Representative images of a PLA positive BC sample (T positive) and its normal counterpart that scored negative in PLA (N negative). Merge of bright field, Red fluorescence and DAPI is reported. Images on the right are the magnification of regions delimited by a yellow square. Scale bar, 20 μm.

Having demonstrated that E-cadherin was delivered to autophagosomes, we then investigated whether SQSTM1/p62 was the cargo adaptor for this process. To this end we took advantage of cells models engineered to express GFP-LC3B (MDA231-GFP-LC3B and HCC1937-GFP-LC3B). E-cadherin, LC3B, and SQSTM1/p62 colocalized in the typical puncta of autophagosomes (Figures 4A,B, arrowheads; Supplementary Figure 6), particularly under Starv+CQ condition. Moreover, under Starv+CQ, both E-cadherin/GFP-LC3B and E-cadherin/SQSTM1/p62 colocalized dots were significantly augmented (Figures 4A,B, right graphs). GPF-positive dots were absent/negligible detected in cells expressing EGFP (Supplementary Figures 5C,D) ruling out that GFP-LC3B puncta were a side-effect of EGFP overexpression.

The interaction between E-cadherin and SQSTM1/p62 was further revealed by proximity ligation assay (PLA) (Weibrecht et al., 2010). The number of PLA puncta were significantly higher in MDA231 cells ectopically expressing E-cadherin (MDA231-E-cad) compared to MDA231-EGFP control cells (Figure 4C). A similar PLA pattern was demonstrated in HCC1937 cells on endogenous E-cadherin (Figure 4D). Interestingly, the amount of PLA puncta per cell significantly increased in HCC1937 cells treated with BAF compared to untreated control consistent with the increment of both E-cadherin and SQSTM1/p62 in these cells (*p* < 0.01, Figure 4D and Supplementary Figure 2C). Samples stained with either anti-SQSTM1/p62 or anti-E-cadherin single antibody and samples incubated with both anti-E-cadherin and anti-GAPDH antibodies, as a negative control, scored essentially negative by PLA whereas in both MDA231-E-cad and HCC1937 cells the assay with anti-E-cadherin and anti-beta-catenin antibodies yielded positive results, as expected (Supplementary Figures 7A,B).

Next, we further sought to validate the SQSTM1/p62-E-cadherin physical interaction by pull-down experiments. To this end, we generated MDA231 cells expressing either E-cadherin or SQSTM1/p62 fused with Strep-Tag and tested these cells in Strep-tactin pull-down experiments. The pull-down of E-cadherin-Strep-Tag allowed the recovery of SQSTM1/p62 (Figure 4E) whereas the reciprocal experiment with SQSTM1/p62-Strep-Tag yielded the recovery of E-cadherin (Figure 4F). Importantly, SQSTM1/p62-E-cadherin binding was confirmed on endogenous proteins by co-immunoprecipitation (IP) in HCC1937 cells (Figure 4G).

Additionally, E-cadherin-SQSTM1/p62 interaction was validated on human primary tumors by performing PLA on a series of E-cadherin positive breast cancers. Colocalization of the two proteins was detected in 20% of tumor samples suitable for the PLA assay (Figure 4H and Supplementary Figure 1B). In tumor sections scoring positive in the PLA assay, normal ducts were PLA negative, hinting that the SQSTM1/p62-E-cadherin colocalization may be a cancer specific phenomenon (Figure 4H).

### SQSTM1/p62 Downregulation Impacts on E-cadherin and LC3B Colocalization

**FIGURE 5:**
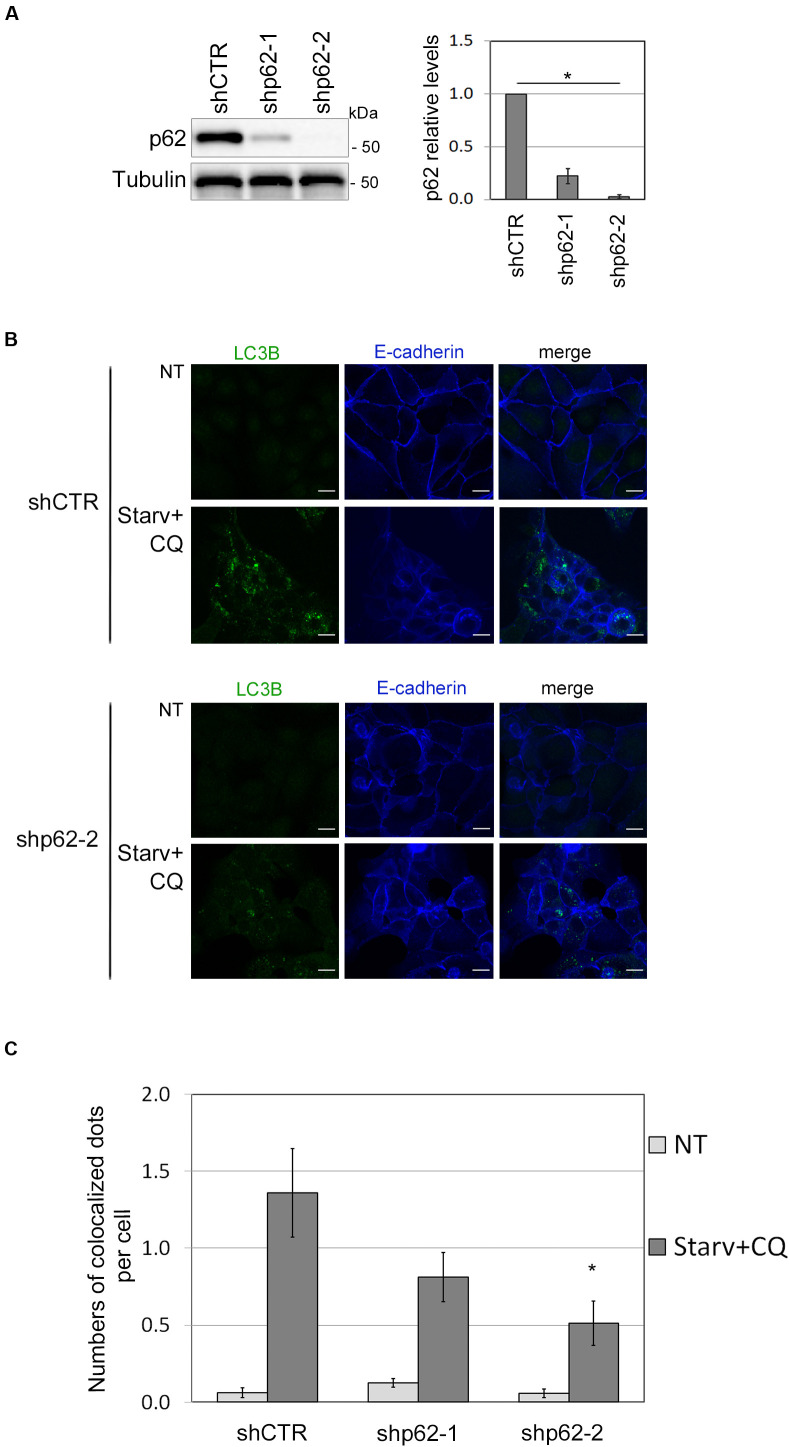
SQSTM1/p62 knock-down restrains the E-cadherin delivery into autophagosomes. **(A)** Immunoblot showing the expression of SQSTM1/p62 in silenced (shp62-1, shp62-2) and in control (shCTR) HCC1937 cell line. The graph on the right reports the mean of SQSTM1/p62 relative levels of three experiments with SEM as error bars. SQSTM1/p62 relative levels were obtained by normalization over γ-tubulin (Tubulin, loading control) and rescaling to the control sample. The asterisk means statistical significance (*p* < 0.001) in the ANOVA test. **(B)** Representative images of shp62-2 and shCTR HCC1937 cell models untreated (NT) or starved and treated with CQ (Starv+CQ) for 8 h showing the E-cadherin and LC3B molecules stained with anti-E-cadherin/Alexa Fluor 633 and anti-LC3B/Alexa Fluor 488 antibodies, respectively. Merge images are reported. Scale bar, 20 μm. **(C)** Graph reported the quantification of LC3B and E-cadherin colocalized dots per cell in the experiments carried out as in **(B)**. At least 100 cells per sample were measured in three independent experiments. Bars show SEM. *p < 0.05.

To further validate the role of SQSTM1/p62 in E-cadherin delivery into autophagosomes, we assessed whether E-cadherin and LC3B colocalized dots decreased by SQSTM1/p62 knock-down. To this end, two different shRNA sequences (shp62-1, shp62-2; Figure 5A) targeting SQSTM1/p62 were transduced by lentiviral infection into HCC1937 and the autophagic process was triggered by Starv+CQ treatment. Knock-down of SQSTM1/p62 expression was associated with a reduction in colocalized E-Cadherin and LC3B dots in the Starv+CQ condition (Figures 5B,C).

## Discussion

Mounting evidence suggests that autophagy modifies the expression of epithelial molecules, affects the integrity of epithelium and impacts on tumor cell motility, invasion, and metastasis (Sharifi et al., 2016).

Here we show that autophagy modulation impinges upon E-cadherin levels and is instrumental in disrupting adherens junctions. Thus, our results broaden the spectrum of cell-to-cell adhesion molecules whose expression is affected by autophagy activation (Fong et al., 2012).

The fact that E-cadherin may be downregulated through the activation of autophagy has been already demonstrated on melanoma and hepatoma cells in which SIRT1 and SPHK1, respectively, regulate this process (Liu et al., 2017; Sun et al., 2018; Zhou et al., 2018). Here we went further by demonstrating that in breast cancer E-cadherin is delivered to autophagosomes. This phenomenon contributes to regulating E-cadherin levels in addition to the previously reported proteasomal and endo/lysosomal degradation pathways (Palacios et al., 2005; Cadwell et al., 2016) and reducing E-cadherin availability at the cell-cell junctions. Notably, the trafficking of E-cadherin to lysosomes has been shown to impinge on the stability of the epithelium and is considered an early event that precedes transcriptional inhibition of CDH1 during epithelial to mesenchymal transition (Palacios et al., 2005).

In addition, we demonstrated that the delivery of E-cadherin to autophagosomes is mediated by the autophagy adaptor SQSTM1/p62. Intriguingly, SQSTM1/p62 was one of the molecules isolated as part of E-cadherin-and F-actin-mediated adherens plaques (Guo et al., 2014).

SQSTM1/p62 has been found to be overexpressed in several cancer types where it induces the expression of inflammatory genes and triggers epithelial to mesenchymal transition, cell proliferation and metastasis (Qiang et al., 2014; Moscat et al., 2016; Puvirajesinghe et al., 2016; Taniguchi et al., 2016). Our data on the role of SQSTM1/p62 in the E-cadherin turnover add a piece of evidence in support of the role of this molecule in these tumor phenotypes.

Further investigations on a larger series of breast cancer might determine the prognostic role of E-cadherin and SQSTM1/p62 interaction and might shed light on the lack of consensus regarding the prognostic value of the sole E-cadherin (Horne et al., 2018).

Interestingly, a recent report involves another autophagy cargo protein, namely NBR1, in breast cancer metastatic dissemination (Marsh et al., 2020). By using different mammary cancer mouse models, the authors show that although genetic ablation of autophagy attenuates primary tumor growth, eventually fuels metastatic outgrowth and that NBR1 plays a key role in this context. These results suggest that the autophagic machinery may participate to tumor inception and progression by relying on different actors.

In conclusion, these findings suggest a model in which autophagy controls E-cadherin turnover through SQSTM1-mediated autophagosome delivery. Thus, the activation of autophagy, which is reported in breast cancers with poor prognosis and in dormant breast cancer cells (Lazova et al., 2012; Vera-Ramirez, 2019), might participate in the control of tumor cells spreading via modulation of E-cadherin.

## Data Availability Statement

The datasets generated for this study are available on request to the corresponding author.

## Ethics Statement

The studies involving human participants were reviewed and approved by the Institutional Review Board of CRO Aviano National Cancer Institute. The patients/participants provided their written informed consent to participate in this study.

## Author Contributions

VD performed the western blots and IP, PD and time-lapse experiments as well as participated in the design of the study and in writing the manuscript. PS gave key ideas for the experiments with confocal laser scanning microscopy and participated in those analyses. GV performed the western blots analyses. TP assessed and selected E-cadherin positive breast tumors. RM discussed the interpretation of data and participated in writing the manuscript. MS designed experiments, did immunofluorescence and PLA studies, analyzed the data, and wrote the manuscript. All authors contributed to the article and approved the submitted version.

## Conflict of Interest

The authors declare that the research was conducted in the absence of any commercial or financial relationships that could be construed as a potential conflict of interest.

## Footnotes

1 https://github.com/ekatrukha/ComDet

## Abbreviations

AJ: Adherens-junctions
BAF: Bafilomycin A1
BC: Breast cancer
CQ: Chloroquine Diphosphate Salt
IP: immunoprecipitation
LC3B: MAP1LC3B
PD: pull-down
PLA: Proximity ligation assay
Rapa: Rapamycin
shRNA: short hairpin RNA
Starv: starvation.
