# Supplementary material for: The Autophagy Machinery Contributes to E-cadherin Turnover in Breast Cancer
Source: Front Cell Dev Biol. 2020 Jun 30;8:545. doi: 10.3389/fcell.2020.00545 (PMC7344152; doi:10.3389/fcell.2020.00545)
Supplement: Supplementary file 1 [file Data_Sheet_1.PDF]

Supplementary Table 1: Primer sequences for lentiviral DNA constructs

Oligo	Sense primer (5'- 3')	Antisense primer (5'- 3')
shCTR	CCGGGCTCCGTGAACGGCCACGAGTGTTAATATTCATAGCACTCGTGGCCGTTCACGGAGCTTTTTT	AATTAAAAAAGCTCCGTGAACGGCCACGAGTGCTATGAATATTAACACTCGTGGCCGTTCACGGAGC
shATG7	CCGGGGAGTCACAGCTCcTCCTTGTTAATATTCATAGCAAGGAAGAGCTGTGACTCCTTTTTT	AATTAAAAAAGGAGTCACAGCTCTTCCTTGCTATGAATATTAACAAGGAgGAGCTGTGACTCC
shp62-1	CCGGCCTCTGGGTATTGAAGTTGGTGTTAATATTCATAGCATCAACTTCAATGCCCAGAGGTTTTTT	AATTAAAAAACCTCTGGGCATTGAAGTTGATGCTATGAATATTAACACCAACTTCAATACCCAGAGG
shp62-2	CCGGGCATTCGTAATGTTGGTTTCAGTTAATATTCATAGCTGAAACCAACATTGCGGATGCTTTTTT	AATTAAAAAAGCATCCGCAATGTTGGTTTCAGCTATGAATATTAACTGAAACCAACATTACGAATGC

Supplementary Table 2: Primary antibodies used in the study

Protein	Species	Type	Reference	Dilution	Supplier
**Western blot, pull-down and IP analyses**					
**E-cadherin**	Mouse	Monoclonal	610182	1/2500	BD Biosciences
**LC3B**	Rabbit	Polyclonal	NB100-2220	1/1000	Novus Biological, CO
**SQSTM1/p62**	Mouse	Monoclonal	sc-28359	1/2000	Santa Cruz Biotechnology, CA
**SQSTM1/p62**	Rabbit	Polyclonal	PA5-27247	1/100	Thermo Fisher Scientific
**IgG1**	Mouse	Isotype Control	02-6100	1/500	Thermo Fisher Scientific
**ATG7**	Rabbit	Monoclonal	8558	1/1000	Cell Signaling Technology, MA
**γ-tubulin**	Mouse	Monoclonal	T6557	1/10000	Sigma-Aldrich, MO
**StrepMAB-Classic**	Mouse	Monoclonal	2-1509-001	1/1000	IBA-Lifesciences
**Immunofluorescence and PLA**					
**E-cadherin**	Rabbit	Monoclonal	3195	1/200	Cell Signaling Technology, MA
**LC3B**	Rabbit	Polyclonal	2775	1/150	Cell Signaling Technology, MA
**SQSTM1/p62**	Mouse	Monoclonal	sc-28359	1/200	Santa Cruz Biotechnology, CA
**β-catenin**	Mouse	Monoclonal	610154	1/200	BD Biosciences
**GAPDH**	Mouse	Monoclonal	sc-32233	1/200	Santa Cruz Biotechnology, CA

A

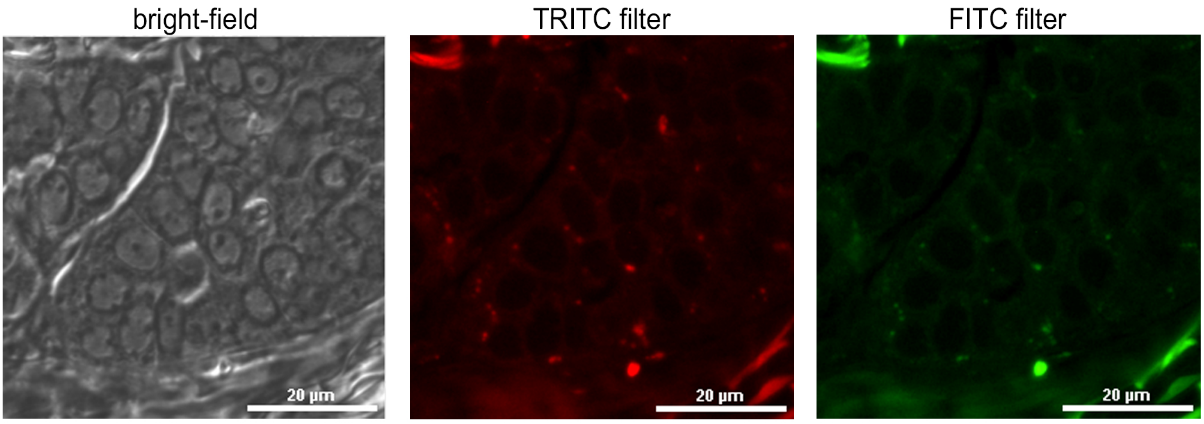

B

	Total (N=19)		Positive PLA (N=3)[a]	Negative PLA (N=12)[a]	*P*
Median age at diagnosis (yrs)	56		37	61	*0.025*[b]
**Tumor size**					*ns*[c]
T1	17		2	11	
T2	2		1	1	
**Tumor grade**					*ns*[c]
G1-G2	9		0	7	
G3	10		3	5	
**Lymph nodes**					*ns*[c]
N neg	14		1	10	
N pos	5		2	2	
**Hormonal receptors**					*ns*[c]
ER/PgR pos	17		3	10	
TNBC	2		0	2	

[a] The sum does not add up to the total because of 4 excluded cases
[b] Mann-Whitney Rank Sum Test
[c] Fisher's exact test

Supplementary Figure 1. Primary infiltrating breast cancers

(A) Representative images of a breast cancer excluded from the PLA analyses for the high level of autofluorescence. Tissue was observed at the epifluorescence Nikon Ti microscope before performing PLA analyses (as described in Materials and Methods). Bright-field and fluorescence images obtained by using either TRITC or FITC fluorescence filter cubes are shown.

(B) The table displays the clinicopathological features of the E-cadherin positive breast cancers according to PLA results.

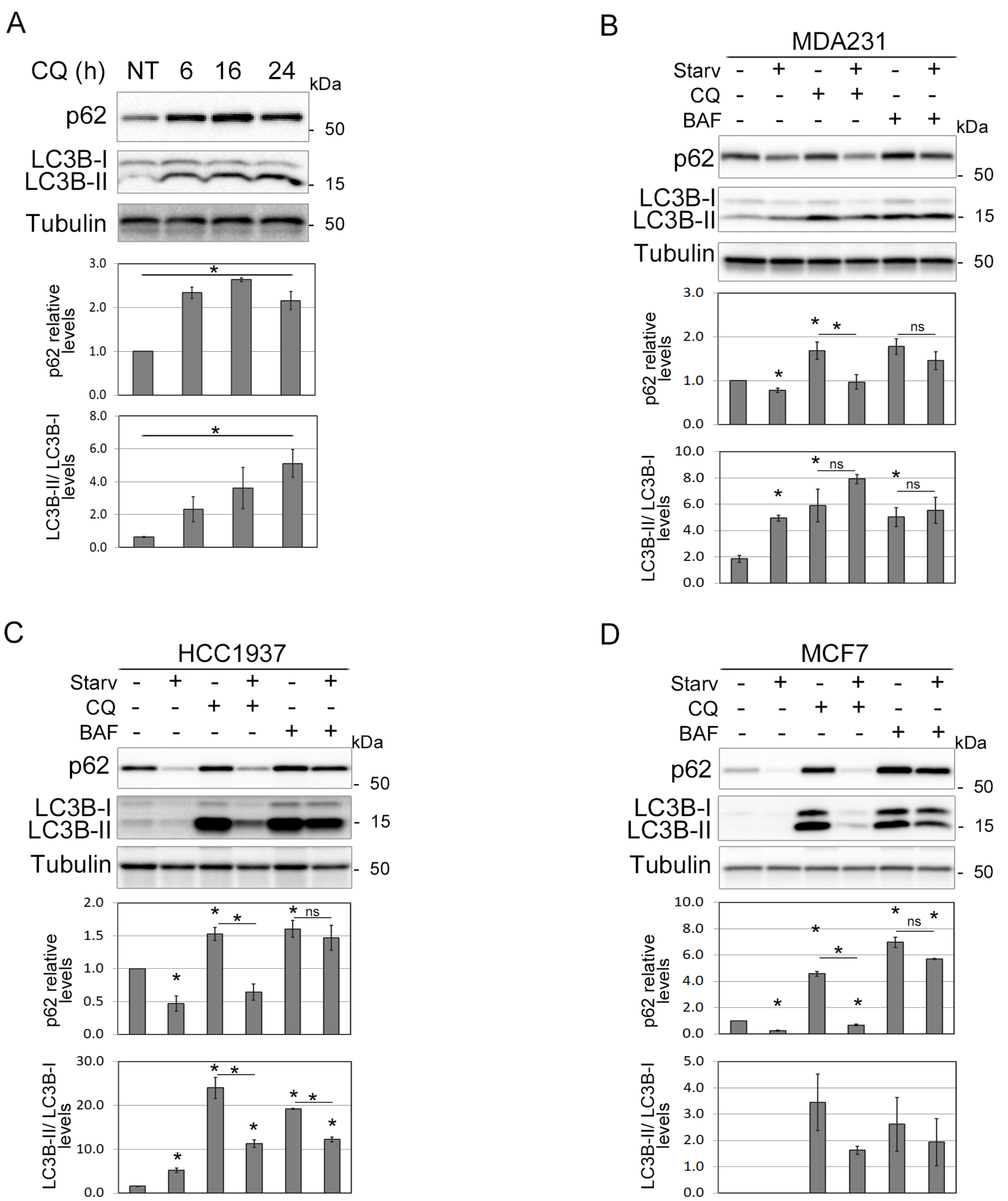

Supplementary Figure 2. Efficacy of cell treatments related to Figure 1

Representative blots of SQSTM1/p62 (p62) and inactive (LC3B-I) and active (LC3B-II) forms of LC3B that demonstrated the efficacy of cell treatments of experiments in Figure 1. Graphs below blots report the mean of SQSTM1/p62 (p62) relative levels or LC3B-II/ LC3B-I ratio of three experiments with SEM as error bars. SQSTM1/p62 (p62) relative levels were obtained by normalization over γ-Tubulin (Tubulin, loading control) and rescaling to the untreated sample.

(A) Related to Figure 1A; MDA231 cells treated with 50μM Cholorquine (CQ) for 6, 16 and 24 hours. * Statistical significance ($p < 0.01$) in the ANOVA test followed by a test for linear trend.

(B, C and D) Linked to Figure 1B, C and D; MDA231, HCC1937 and MCF7 cell lines starved (Starv), treated with Cholorquine (CQ) or Bafilomicyn A1 (BAF) etiher in standard medium or in starvation. In untreated and starved MCF7 cells, LC3B is barely detectable in our experimental conditions not allowing protein level quantification. The asterisks above the histograms and lines mean statistical significance ($p< 0.05$; unpaired *t*-test) in comparisons between treaed and untreated samples or between indicated samples, respectively. ns, not statistically significant.

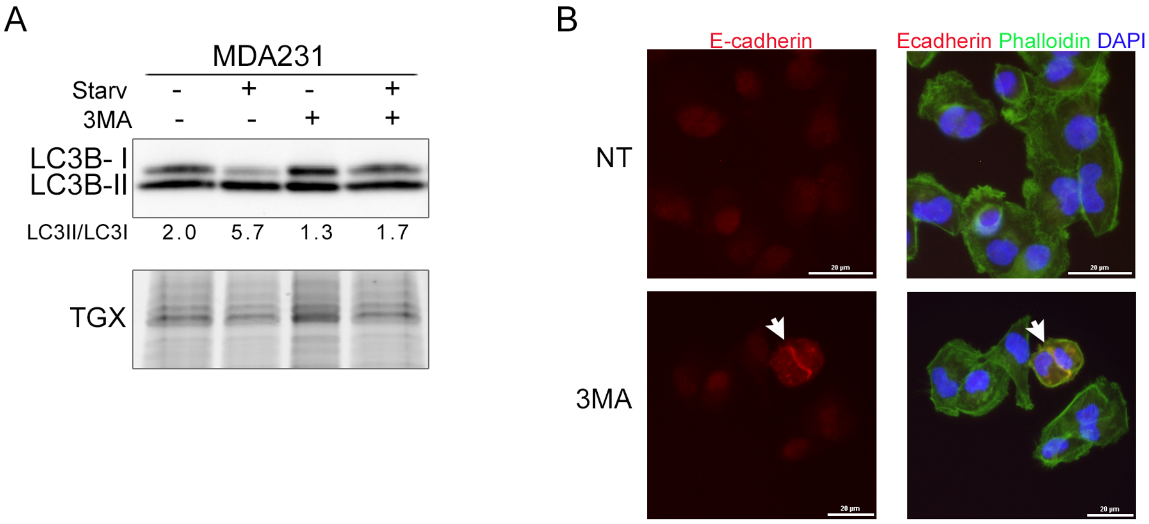

Supplementary Figure 3. E-cadherin localizes at the plasmamembrane in MDA231 cell line treated with 3-methyladenine (3MA), an inhibitor of LC3B activation

(A) Immunoblot showing the expression of the inactive (LC3B-I) and active (LC3B-II) forms of LC3B in MDA231 cells treated for 100 minutes with 3MA 5mM either in standard medium or upon nutrient starvation. The ratio between active and inactive LC3B forms is reported (LC3BII/LC3BI). The efficacy of 3MA treatment is demonstrated by the increment in the inactive LC3B form in 3MA treated cells cultured either in standard medium or in nutrient starvation. Fluorescent emission of stain-free gel (TGX) was reported as a loading control.

(B) Representative images of MDA231 cells either treated for 100 minutes with 5mM 3MA or not treated as a control (NT) and stained with anti-E-cadherin antibody (red), phalloidin488 (green) and DAPI (blue). White arrows indicate E-cadherin at the plasma membrane.

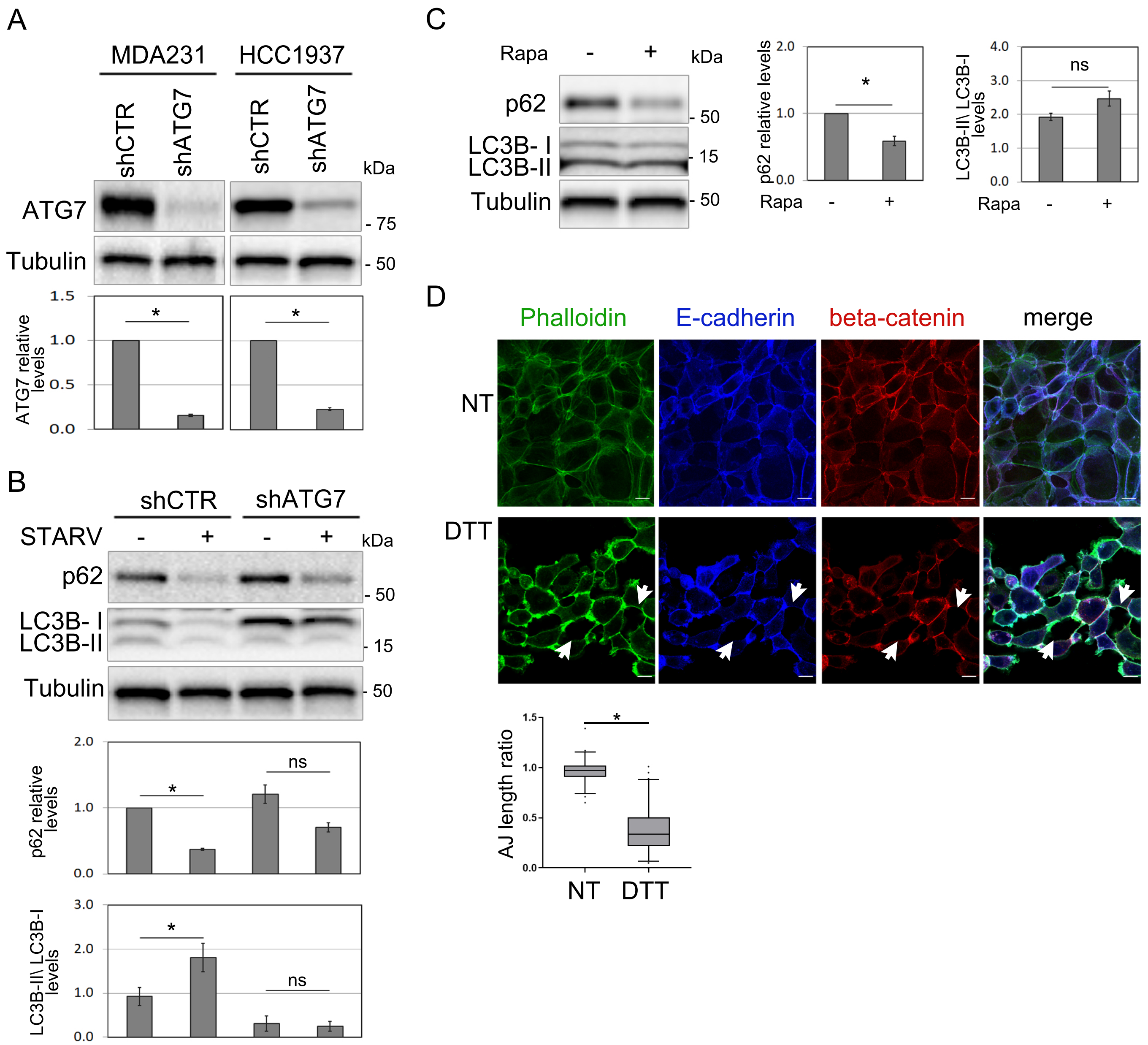

Supplementary Figure 4.

(A) ATG7 silencing in the MDA231 and HCC1937 cell lines. Immunoblots showing the expression of ATG7 and γ-Tubulin (Tubulin) used as a loading control, in silenced (shATG7) and control (shCTR) cells. Graphs below blots report the mean of ATG7 relative levels obtained by normalization over Tubulin. The asterisks mean statistical significance ($p < 0.01$) assessed by the unpaired *t*-test.

(B) Representative immunoblots of SQSTM1/p62 (p62) and the inactive (LC3B-I) and active (LC3B-II) forms of LC3B (lower panel) in control (shCTR) and in ATG7-silenced (shATG7) HCC1937 cells either untreated (NT) or starved (STARV). γ-Tubulin (Tubulin) was used as a loading control.

(C) Immunoblots showing the expression of SQSTM1/p62 (p62) and the inactive (LC3B-I) and active (LC3B-II) forms of LC3B in the HCC1937 cell line treated with 5 μM Rapamycin (Rapa) for 24 hours.

In **(B)** and **(C)** graphs report the mean of SQSTM1/p62 (p62) relative levels and LC3B-II/ LC3B-I ratio of at least two indipendent experiments with SEM as error bars. SQSTM1/p62 (p62) relative levels were obtained by normalization over γ-Tubulin and rescaling to the untreated sample. The asterisks mean statistical significance ($p < 0.05$) in the unpaired *t*-test. ns, not statistically significant.

(D) Confocal fluorescence images of HCC1937 untreated (NT) or treated with DTT and immunostained with Alexa Fluor 488 Phaolloidin, E-cadherin/Alexa Fluor 633 and beta-catenin/Alexa Fluor 594. White arrowheads indicate representative lack of cell-to-cell cohesion regions. Scale bar, 20 μm. The boxplots represent the quantification of adherens junctions ratio (measured as described in Materials and Methods). Lines within the boxes mark the median, boundaries represent the 25th and the 75th percentiles, whiskers below and above the boxes indicate the 5th and 95th percentiles, respectively, and dots the outliers. The asterisk means statistical significance ($p < 0.0001$) assessed by the unpaired *t*-test in comparisons between the untreated and treated samples

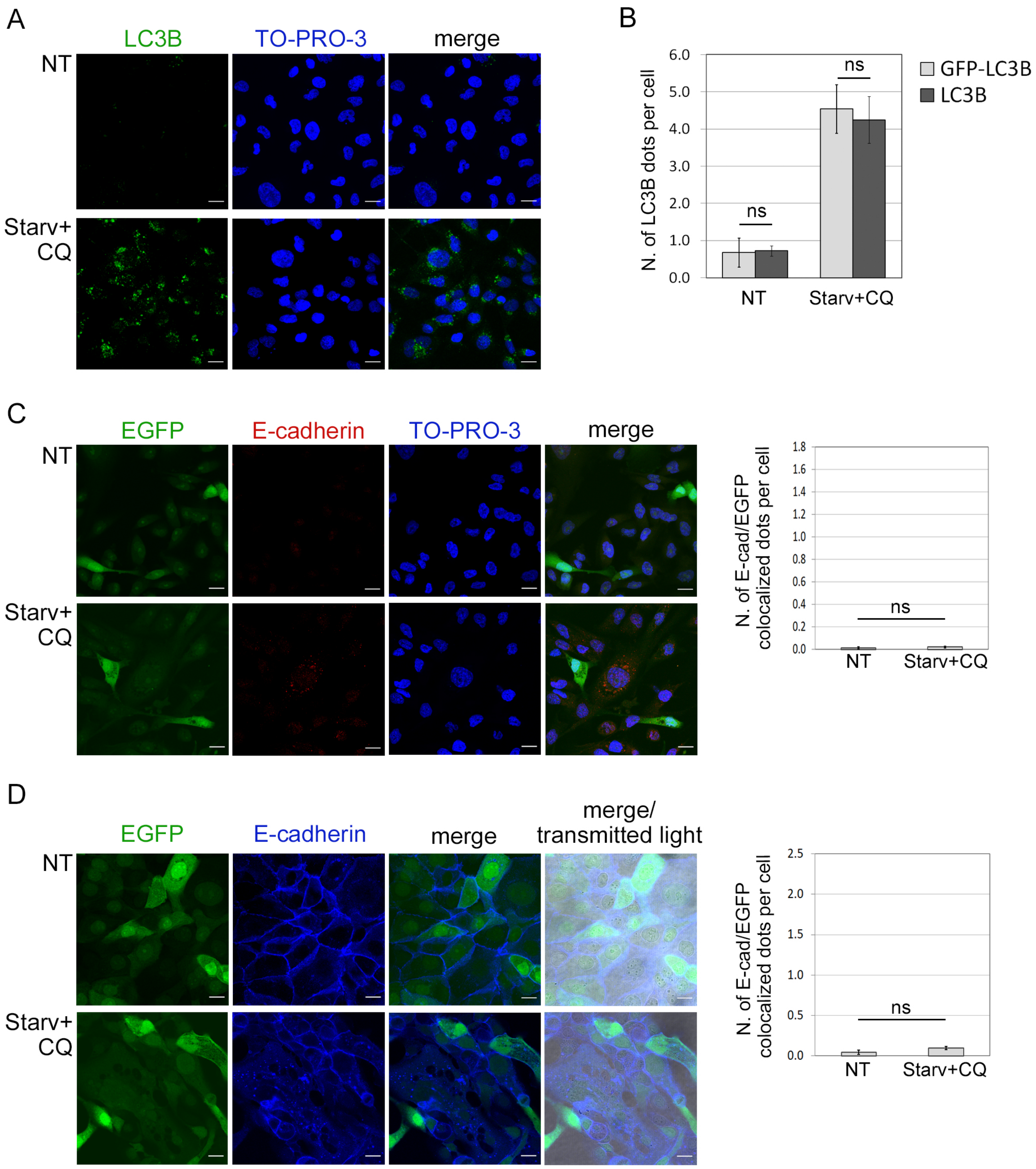

Supplementary Figure 5. (Related to Figure 3).
(A) Representative images of MDA231cell line untreated (NT) or starved in the presence of chloroquine (Starv+CQ) for 8 hours and immunostained for endogenous LC3B. Nuclei were labeled with TO-PRO-3 fluorescence dye. Scale bar, 20 μm.
(B) Quantification of LC3B puncta per cell in MDA231 treated as in (A) and in MDA231 ectopically expressing GFP-LC3B showed as in Figure 3A.
(C) Confocal fluorescence images of MDA231-EGFP untreated (NT) or starved and treated with chloroquine (Starv+CQ) for 8 hours. Cells were immunostained with E-cadherin/Alexa Fluor 594 and TO-PRO-3 nuclear dye. Scale bar, 20 μm. The graph on the right shows the numbers of E-cadherin and EGFP colocalized dots per cell on the same scale as for Figure 3B.
(D) Representative images of HCC1937-EGFP cells untreated (NT) or starved and treated with chloroquine (Starv+CQ) for 8 hours. Cells were immunostained with E-cadherin/Alexa Fluor 633. Merged fluorescent images and transmitted light images were also reported. Scale bar, 20 μm. The graph on the right shows the numbers of E-cadherin and EGFP colocalized dots per cell on the same scale as for Figure 3D.
For graphs (B- D), statistical significance was calculated performing unpaired t-tests. ns, not statistically significant.

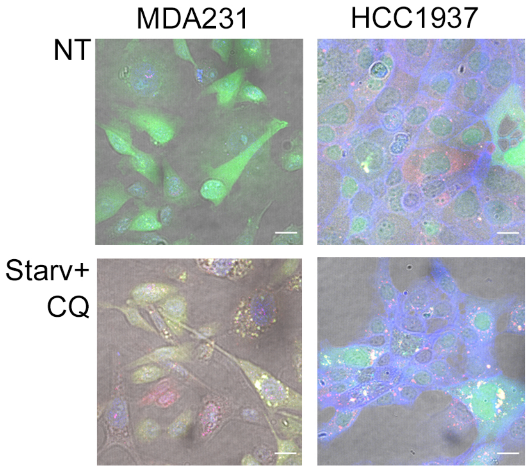

Supplementary Figure 6 (Related to Figure 4A).

Merge of transmitted light and fluorescence images corresponding to images displayed in Figure 4A and B. MDA231 and HCC1937 cell lines were untreated (NT) or starved in the presence of chloroquine (Starv+CQ) for 8 hours. Scale bar, 20 µm.

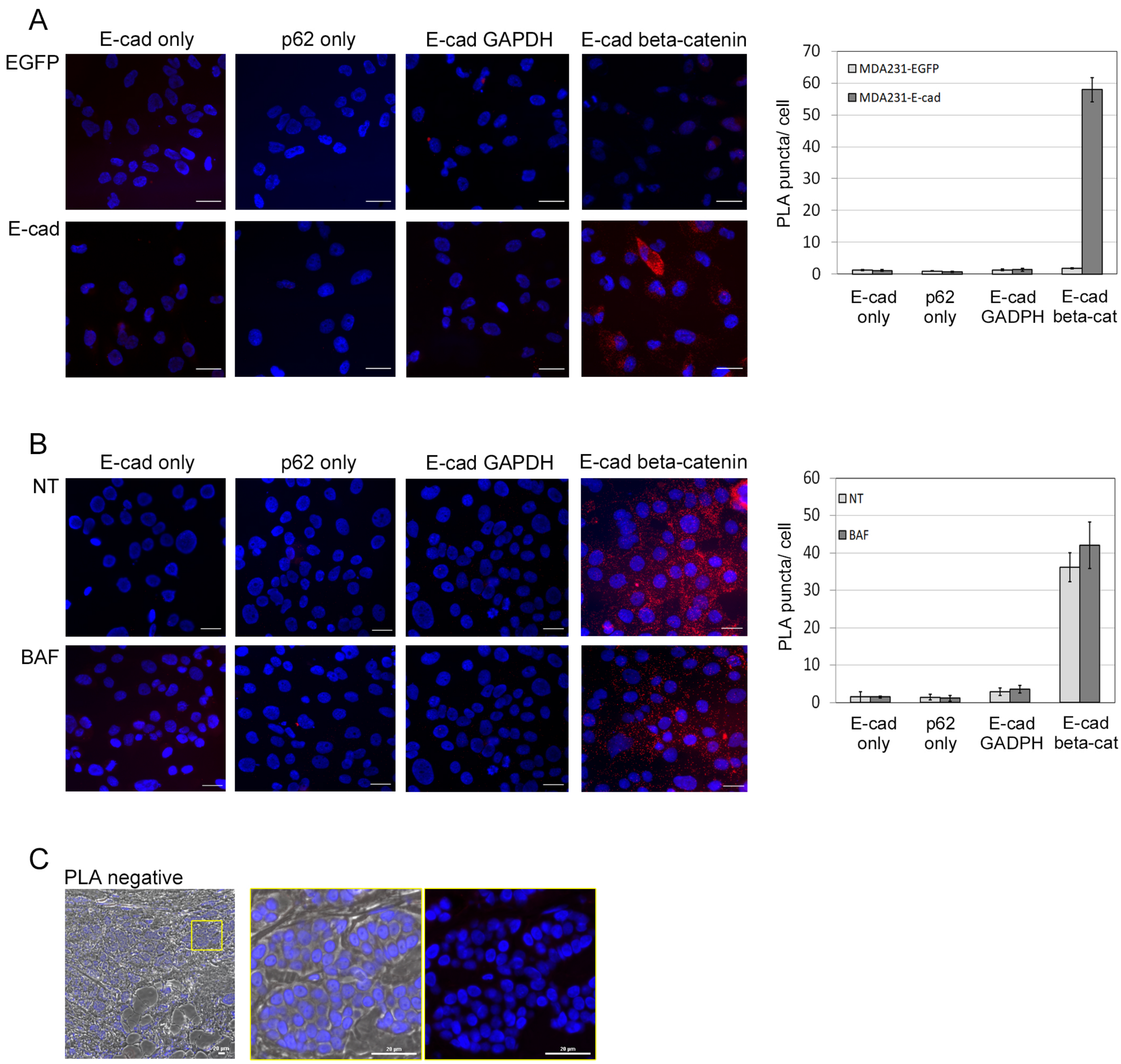

Supplementary Figure 7. Proximity ligation assay (PLA) controls related to Figures 4C, D and H.

(A) Representative images of the MDA231 cells ectopically expressing EGFP (MDA231-EGFP) or E-cadherin (MDA231-E-cad) processed for PLA with single antibody anti-E-cadherin (E-cad only), anti-SQSTM1/p62 (p62 only) and with both anti-E-cadherin and anti-GAPDH (negative control) or anti-E-cadherin and anti-beta-catenin (positive control). PLA foci were barely detectable in all samples but in the anti-E-cadherin/beta-catenin staining of MDA231-E-cad.

(B) Representative images of HCC1937 cell line untreated (NT) or treated with Bafilomycin A1 (BAF) for 8 hours and evaluated for PLA with antibody anti-E-cadherin (E-cad only), anti-SQSTM1/p62 (p62 only) and with both anti-E-cadherin and anti-GAPDH (negative control) or anti-E-cadherin and anti-beta-catenin (positive control). As aspected, PLA foci were essentially negative in all samples but in the anti-Ecadherin/beta-catenin samples.

In **(A** and **B)**, graphs on the right display the quantification of PLA puncta/ cell.

(C) Representative images of a breast cancer scored negative for PLA.
