# Supplementary material for: The Autophagy Machinery Contributes to E-cadherin Turnover in Breast Cancer
Source: Front Cell Dev Biol. 2020 Jun 30;8:545. doi: 10.3389/fcell.2020.00545 (PMC7344152; doi:10.3389/fcell.2020.00545)
Supplement: Supplementary file 2 [file Data_Sheet_2.PDF]

Figure 1A_MDAMB231

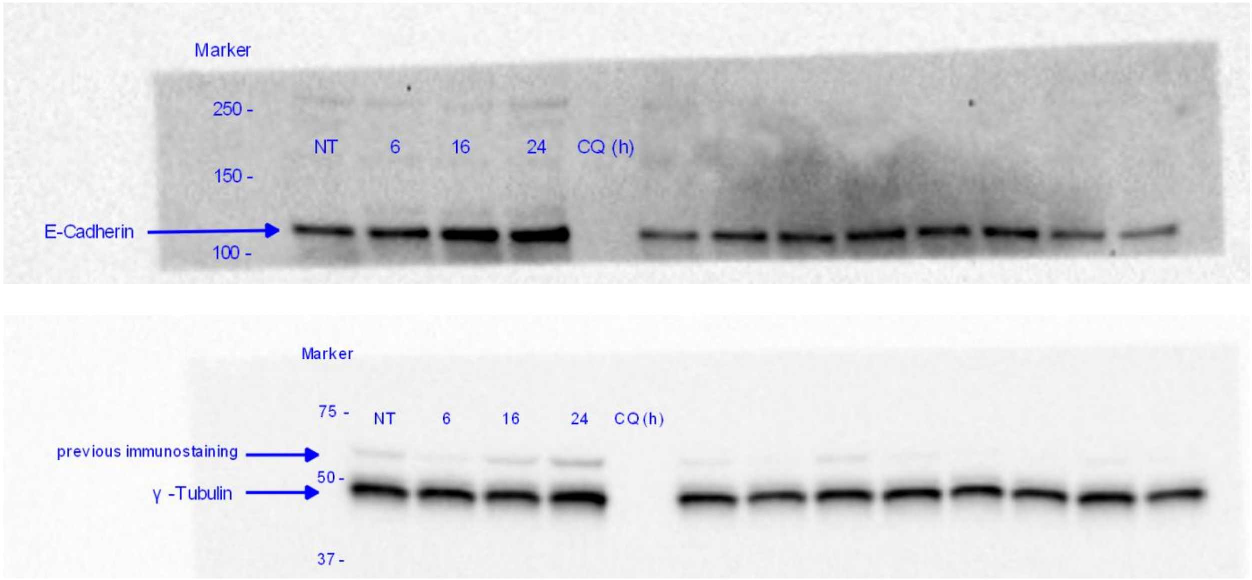

Figure 1B_MDAMB231

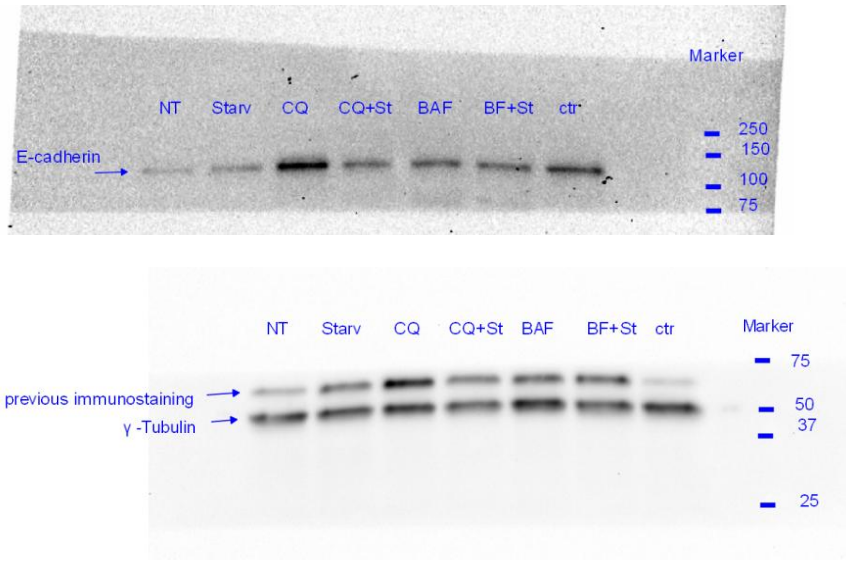

Figure 1C_HCC1937

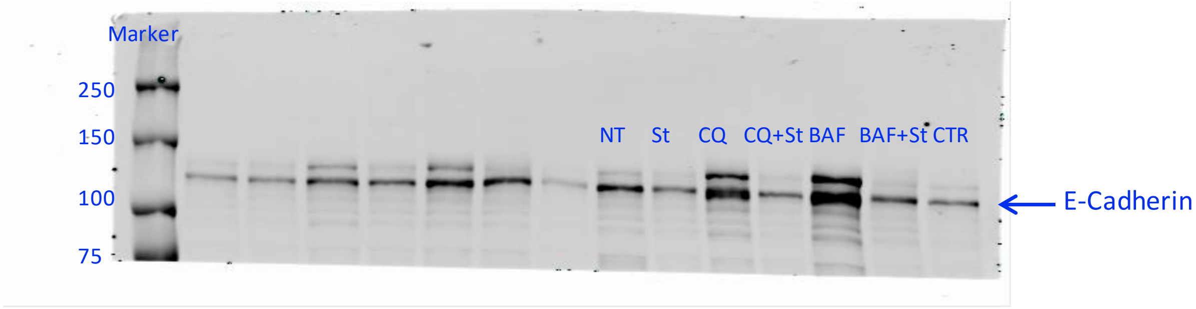

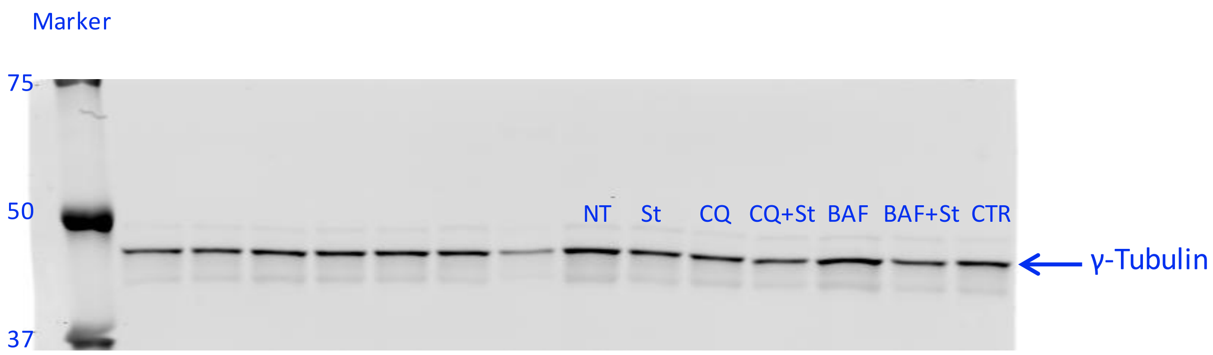

Figure 1D_MCF7

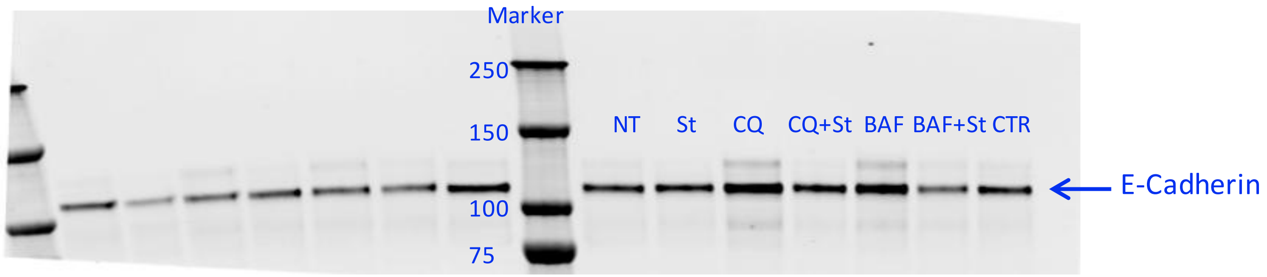

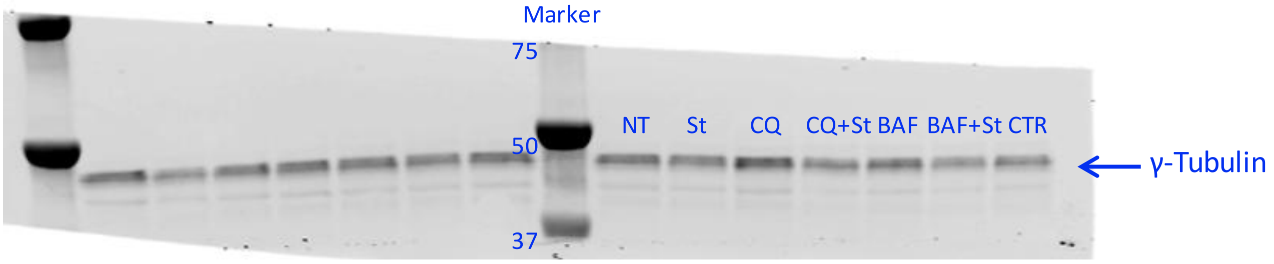

Figure 4D_MDAMB231

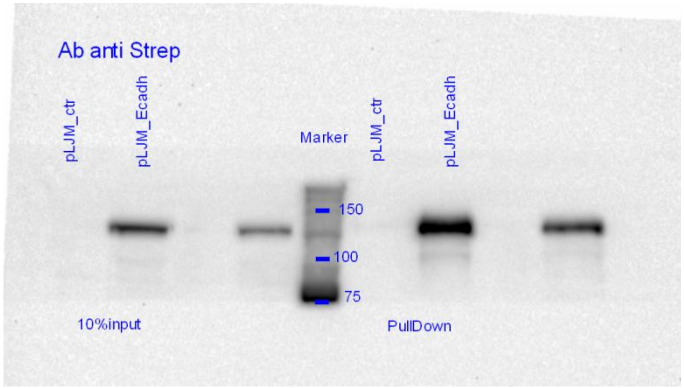

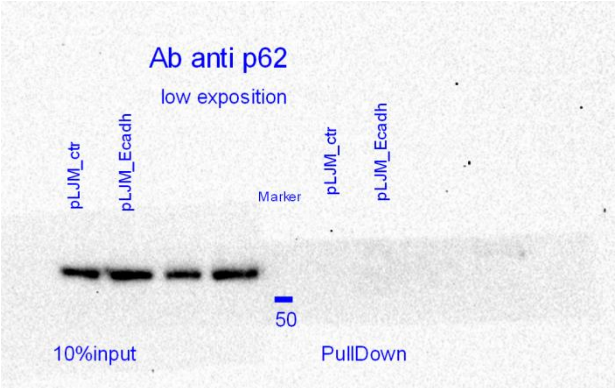

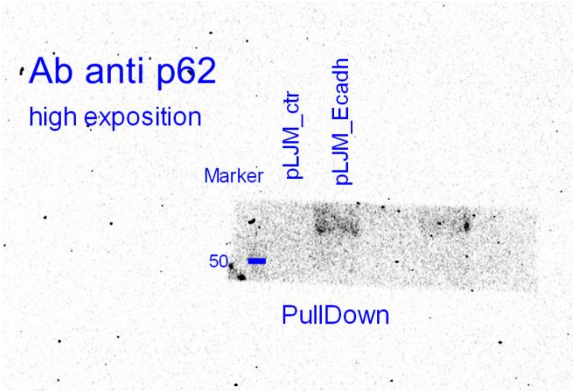

Figure 4E_MDAMB231

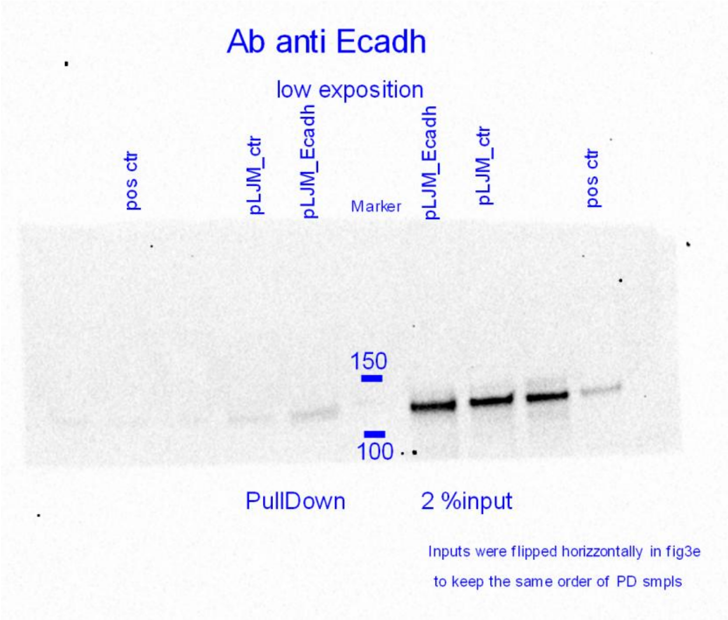

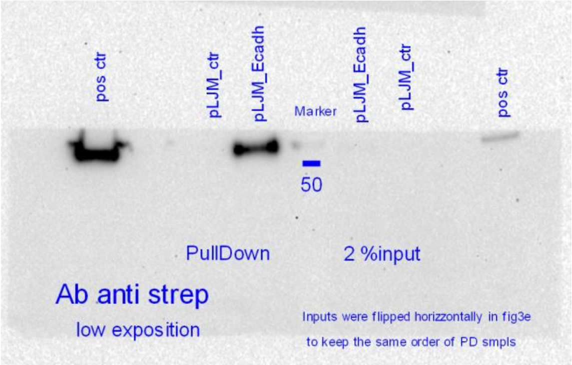

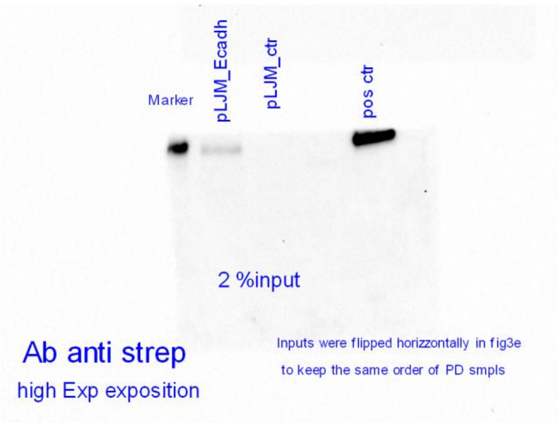

Figure 4F_HCC1937

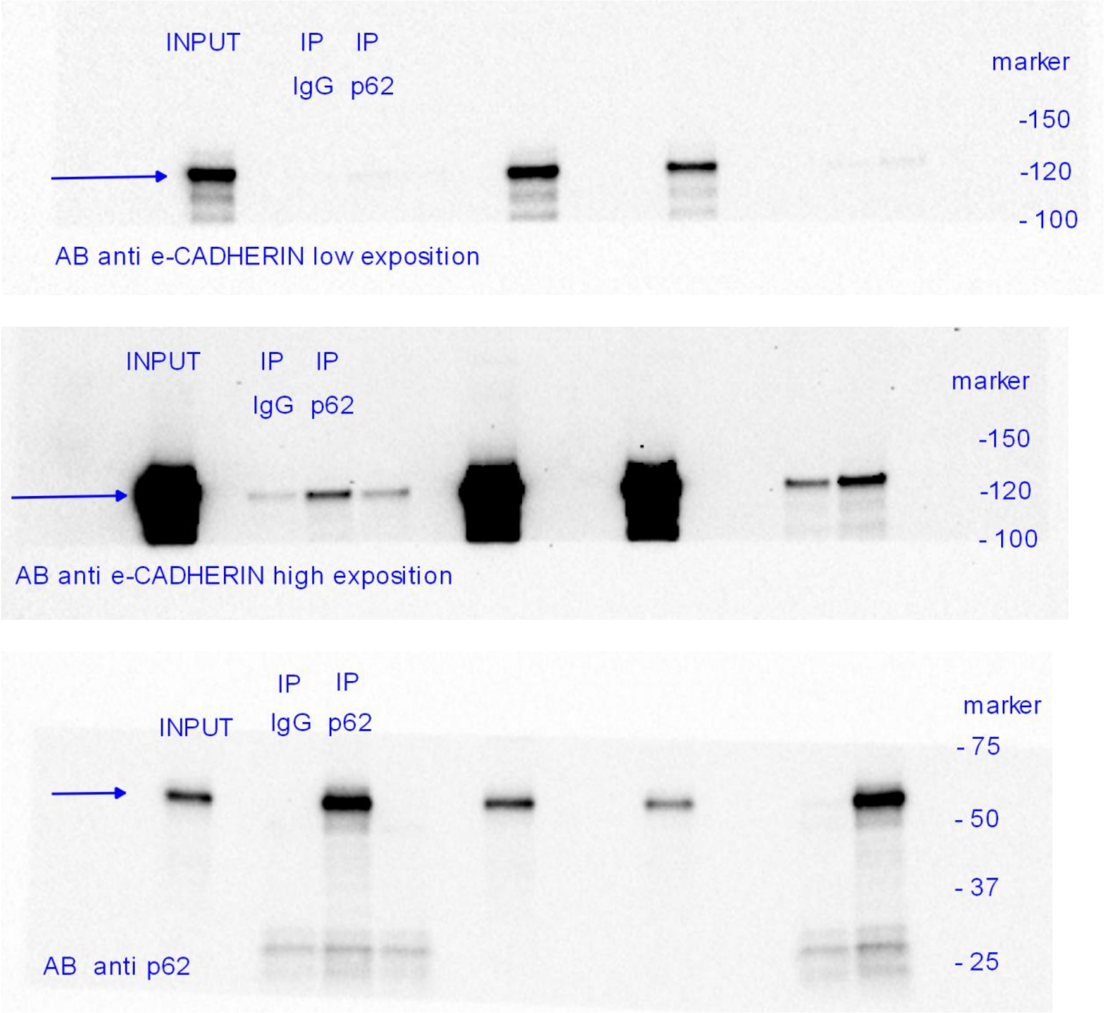

Supplementary Figure S2A_MDAMB231

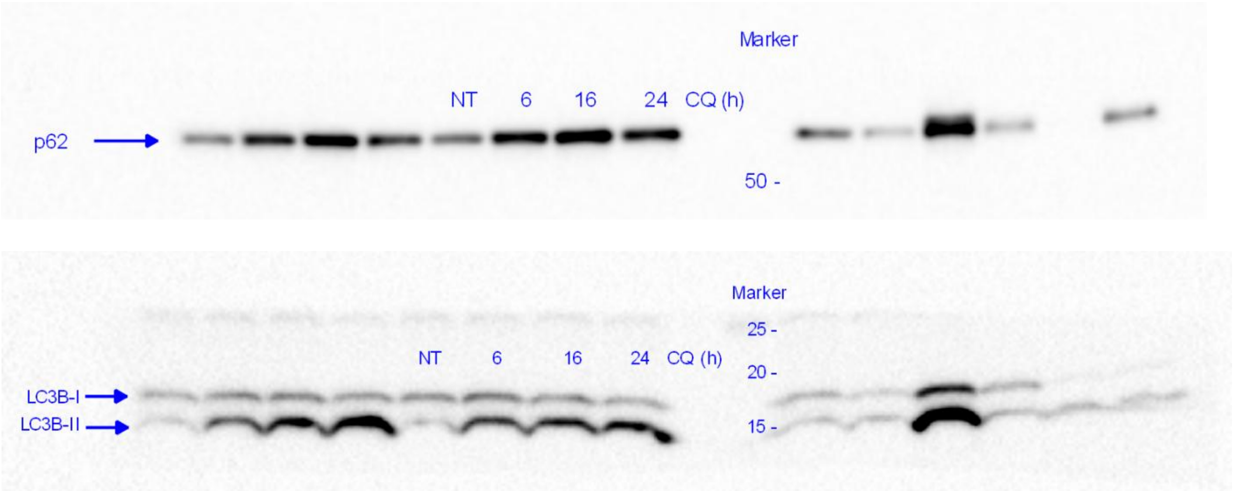

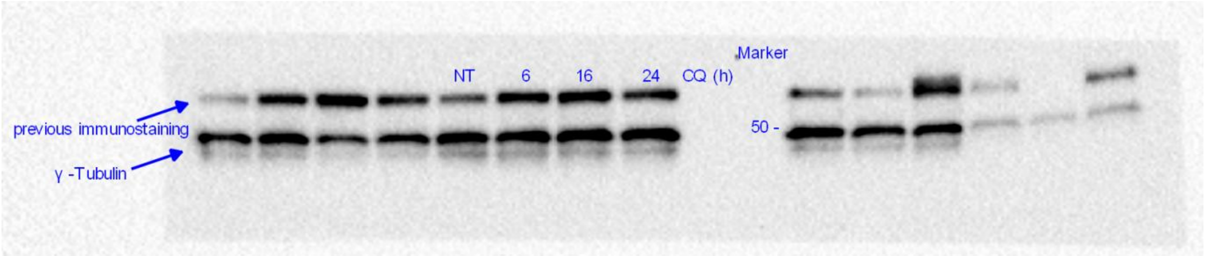

Supplementary Figure S2B_MDAMB231

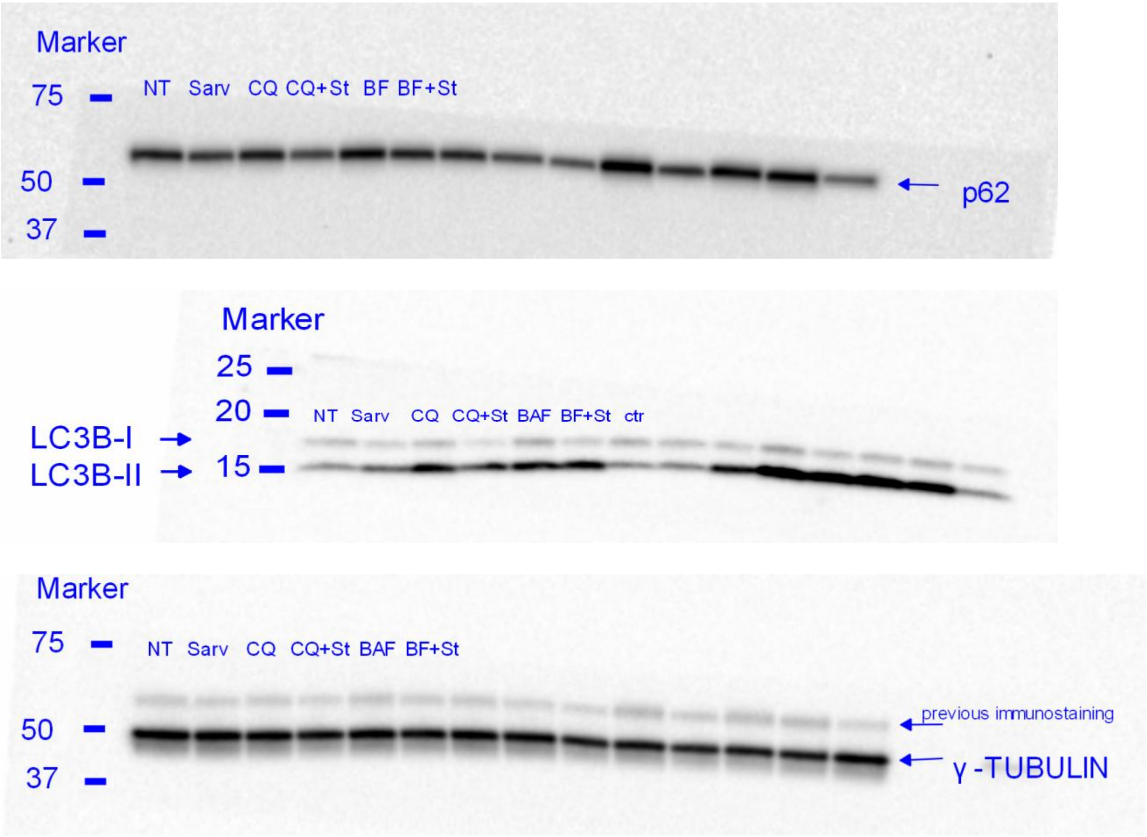

Supplementary Figure S2C_HCC1937

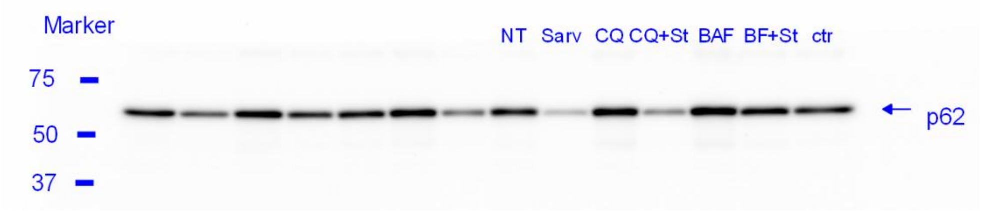

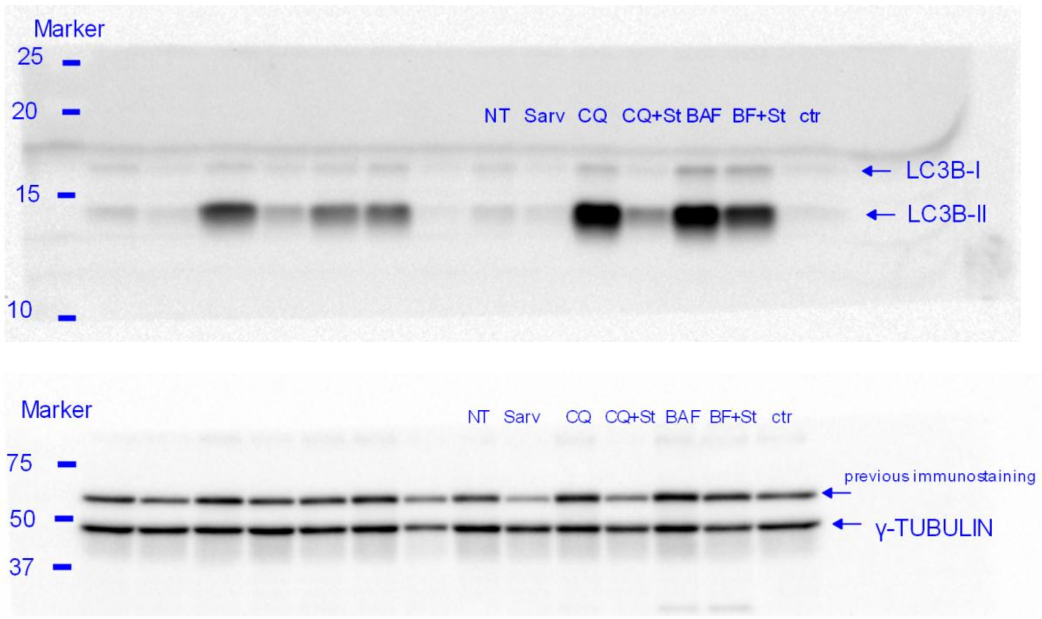

Supplementary Figure S2D_MCF7

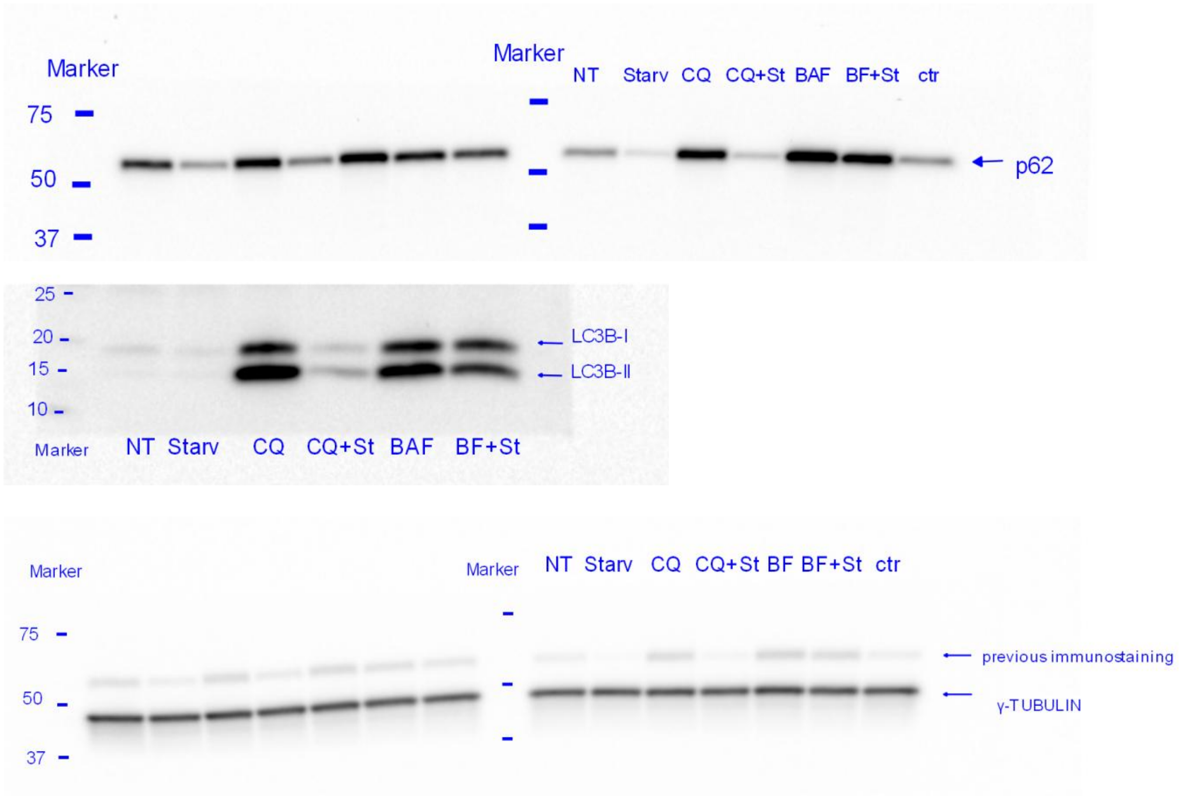

Supplementary Figure 3A_ MDAMB231

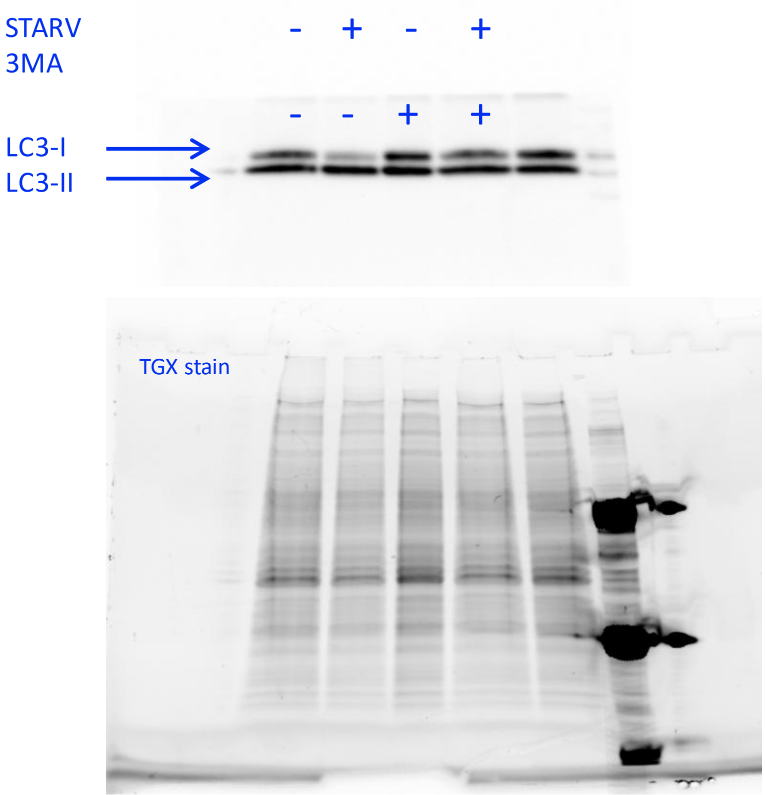

Supplementary Figure 4A_MDA231 and HCC1937 ATG7-silenced cells

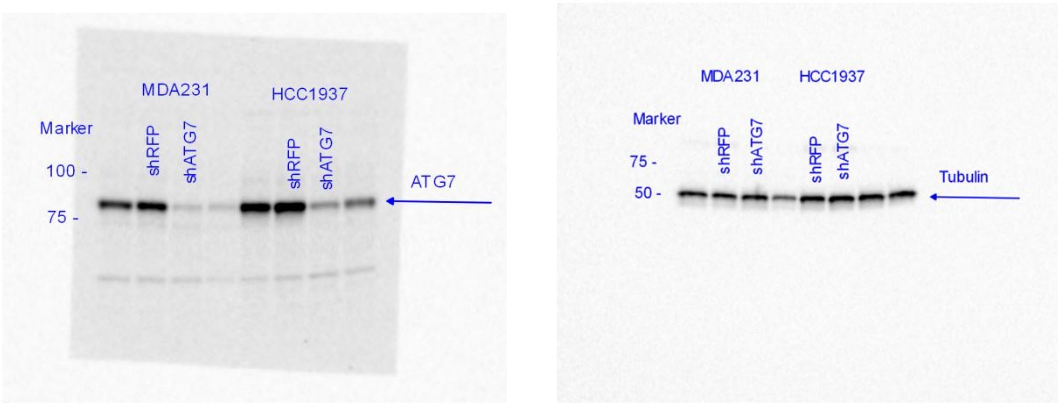

Supplementary Figure 4B_ HCC1937 ATG7-silenced cells

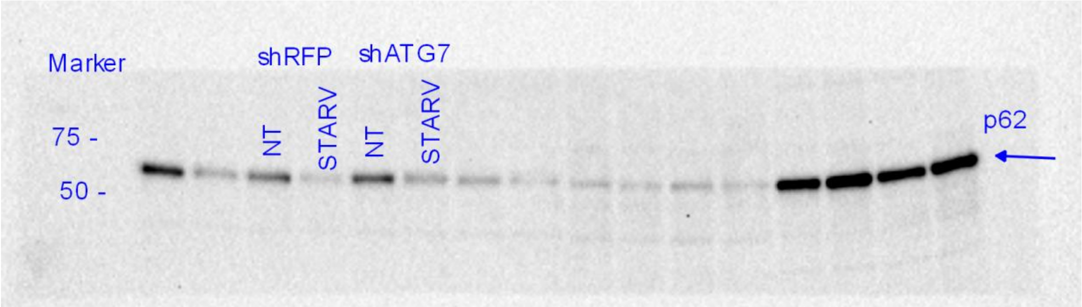

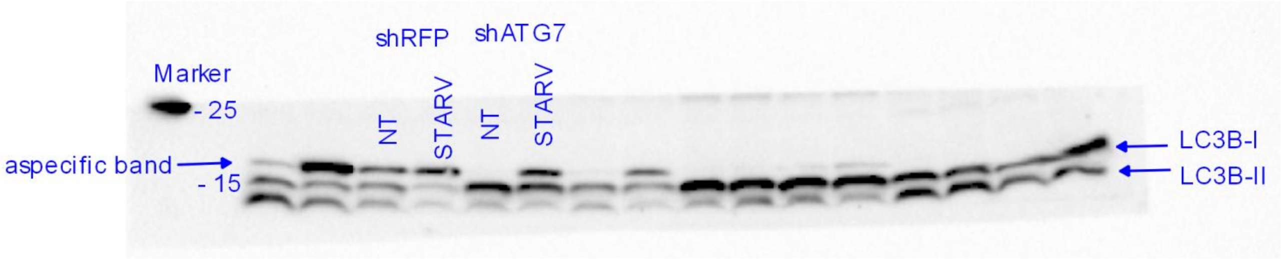

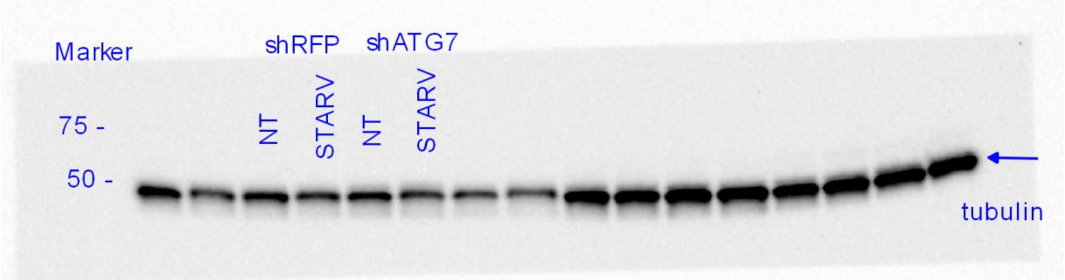

Supplementary Figure 4C_HCC1937 ATG7-silenced cells

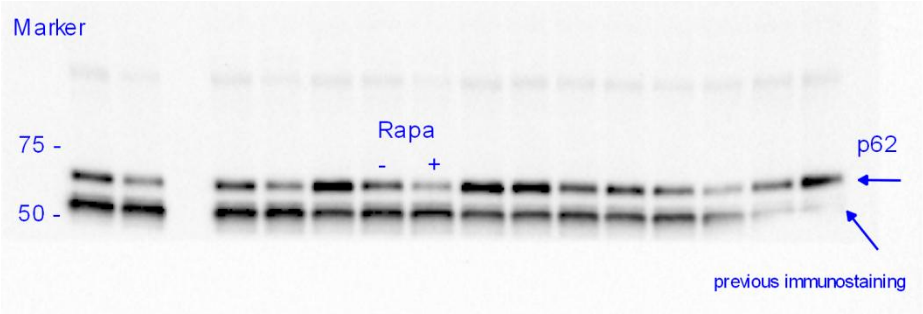

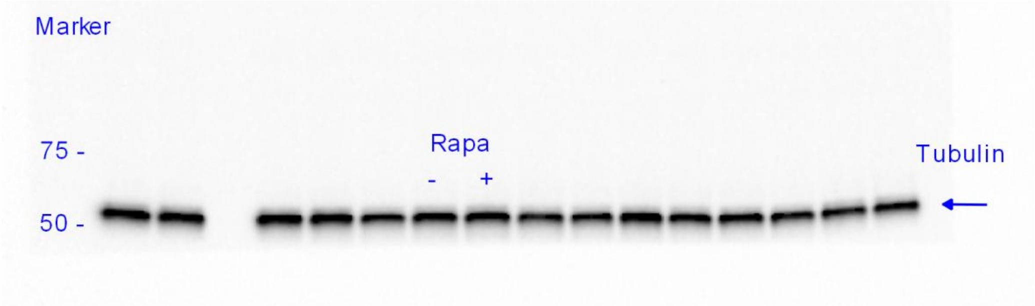

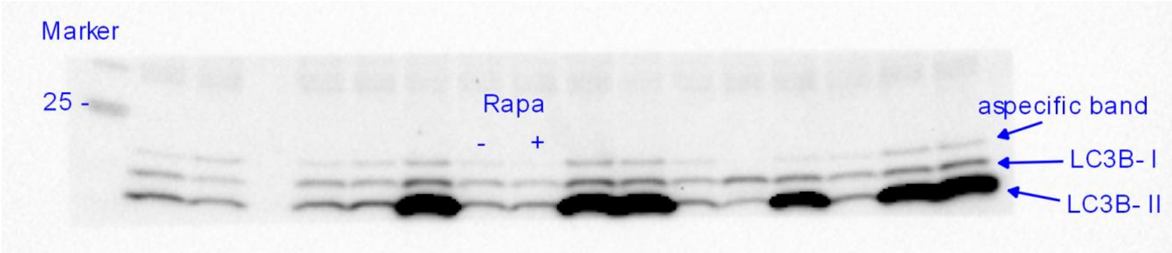
Marker
25 -
Rapa
-
+
aspecific band
LC3B- I
LC3B- II
